# Research progress of implantable materials in antibacterial treatment of bone infection

**DOI:** 10.3389/fbioe.2025.1586898

**Published:** 2025-07-16

**Authors:** Binqing Xiang, Jingui Jiang, Heng Wang, Lei Song

**Affiliations:** ^1^Department of Surgical Anesthesia, First Affiliated Hospital, Army Medical University, Chongqing, China; ^2^ Basic Medical College, Army Medical University, Chongqing, China; ^3^First Affiliated Hospital, Army Medical University, Chongqing, China; ^4^Department of Orthopaedics, First Affiliated Hospital, Army Medical University, Chongqing, China

**Keywords:** bone infection, implantable materials, antibacterial treatment, composite materials, material matrix

## Abstract

Bone infection is an infectious disease characterized by the accumulation of various pathogens in bone tissue, often causing significant suffering to patients. Current therapeutic approaches for bone infections predominantly rely on the postoperative application of implantable antibacterial materials, highlighting their essential role in clinical treatment. In this review, we systematically analyze research progress in antibacterial implant materials for Bone infection from 2019 to 2025. Materials are classified into four categories based on matrix composition: metal-based composite implants, bioceramic-based composite implants, polymer-based composite implants, and other composite implant materials, with dedicated focus on the limitations of each material type. The deterioration effects of these materials are also thoroughly analyzed. Finally, we present our own insights regarding future development directions of antibacterial implant materials. This review aims to provide practical references and research perspectives for advancing antibacterial implant material development.

## 1 Introduction

Bone infection represents a devastating condition characterized by bacterial or other pathogenic invasions into bone tissue, often leading to bone destruction and subsequent bone defects (Porrino et al., 2020). Severe trauma, such as open fractures of extremities, carries a 30% probability of progressing to infectious osteomyelitis, if prompt debridement is not performed, frequently resulting in substantial patient suffering (Metsemakers et al., 2017). The incidence of secondary bone defects caused by surgical resection (e.g., radical tumor excision) has also been increasing (Driscoll et al., 2020; Pazarçeviren et al., 2020). In recent years, with the widespread adoption of joint replacement surgeries, implant-related infections have shown a year-on-year rise, emerging as one of the primary complications within 3 months postoperatively for both knee and hip arthroplasty, and contributing to elevated mortality rates (Rennert-May et al., 2018; Scialla et al., 2021).

According to previous epidemiological investigations, *Staphylococcus aureus* (*S. aureus*) remains the predominant pathogen in bone infections (Kong et al., 2022), Oxacillin-resistant *S. aureus* (MRSA) produces lethal toxins and exhibits high drug resistance, accounting for over 50% of *S. aureus*-related infections (Dudareva et al., 2019). Other pathogens, including *Escherichia coli* (*E. coil*), *Cutibacterium acnes*, and *Pseudomonas aeruginosa*, can also induce bone infections. *S. aureus*-mediated bone infections primarily involve four mechanisms: intracellular infection, osteocyte lacuna-canalicular network (OLCN) invasion, biofilm formation, and abscess development (Libraty et al., 2012; de Mesy Bentley et al., 2017) (detailed in Section 2). These mechanisms collectively enable *S. aureus* to colonize and persist within bone tissue, resulting in protracted osteomyelitis. Consequently, *S. aureus*-induced bone infections are particularly severe and clinically challenging to manage.

Current therapeutic approaches for bone infections primarily involve surgical intervention and antimicrobial administration. Surgical techniques are based on classical Ilizarov and Masquelet methods (Hatashita et al., 2021; Szelerski et al., 2021). Both of which present limitations including joint stiffness, delayed union, or nonunion at defect sites (Aktuglu et al., 2019). Concurrently, the rising bacterial minimum inhibitory concentration (MIC) compromises the efficacy of locally or systemically administered antibiotics (Satola et al., 2011; Aljohani et al., 2020). Consequently, contemporary treatment protocols combine surgical debridement with localized application of antibacterial materials and adjunctive systemic antibiotic therapy. This underscores the critical importance of advanced antibacterial materials in bone infection management, representing both a pivotal strategy for therapeutic improvement and an emerging focus in this field (Ding et al., 2022). Currently approved orthopedic antibacterial implants include: antimicrobial metal-coated implants (e.g., those containing bioactive metals like silver (Ag), zinc (Zn), copper (Cu)); absorbable magnesium (Mg) alloy antibacterial screws; antibiotic-loaded bone cement (gentamicin- polymethyl methacrylate (PMMA)cement); bioceramic composites; and dynamically responsive smart implants. Most of these materials are composite systems, as single-type matrix materials typically exhibit both advantages and limitations. Combining multiple materials (e.g., Mg alloy/graphene oxide (GO)/hydroxyapatite (HAP) composite coatings that inhibit bacterial adhesion through synergistic effects between magnesium hydroxide and GO (Yuan et al., 2022)), enables enhanced functionality, such as smart antibacterial materials capable of autonomously regulating local temperature and pH levels (Frazar et al., 2020; Li et al., 2020; Nagase, 2021). These systems achieve optimal transitions between bactericidal activity and sustained release during different infection phases. Material modifications also address historical limitations: PMMA remains a clinically established bone filler, yet its polymerization process generates substantial heat, imposing thermal stability requirements on incorporated antibiotics (Lai et al., 2013). This exothermic reaction typically causes burst antibiotic release rather than sustained antimicrobial action. To overcome this, researchers have developed PMMA composites incorporating silica particle-gentamicin complexes, achieving prolonged antibiotic release post-implantation (Al Thaher et al., 2018). Surface modifications and doping techniques have been extensively applied to inert metal implants (predominantly titanium [Ti] implants) (Yu et al., 2023; Hou et al., 2025; Tan et al., 2025), alongside emerging materials controllable via photoelectric, photothermal, and magnetic field effects. The antibacterial mechanisms of materials can be broadly categorized into bactericidal and bacteriostatic effects, both contributing positively to bone infection management. Antimicrobial efficacy is achieved through two primary pathways: direct antimicrobial action from material properties, or indirect immune cell modulation via material-tissue interactions. Regardless of the antimicrobial approach employed, effective treatment necessitates defect filling to restore bone continuity, making the selection of appropriate implant matrices critical. Current implant matrices predominantly utilize inert materials, classified as: metal-based composite implants, bioceramic-based composite implants, polymer-based composite implants, and other composite systems. Following this classification framework, we review representative advancements in antibacterial implant materials, analyze associated degenerative issues, and propose countermeasures, aiming to provide systematic references for current research progress in bone infection therapeutic materials.

## 2 Mechanisms of bacterial-induced bone infection

Using *S. aureus* as the primary example, most current implantable antibacterial materials target this pathogen. Understanding its cellular infection mechanisms is essential for rational material design. *S. aureus* can persistently survive intracellularly within macrophages (Garzoni and Kelley, 2011), keratinocytes (Kintarak et al., 2004), epithelial cells, and endothelial cells (Dziewanowska et al., 1999; Edwards et al., 2010). Macrophages harboring intracellular *S. aureus* are termed “Trojan horse” macrophages, which critically facilitate infection dissemination and enrichment of small colony variants (SCVs) (Garzoni and Kelley, 2011). SCVs, a specialized *S. aureus* phenotype, are primarily responsible for chronic recurrent infections. Within bone tissue, *S. aureus* demonstrates persistent survival in osteocytes, osteoclasts (OCs), and osteoblasts (OBs) (Josse et al., 2015; Yang et al., 2018; Krauss et al., 2019). Direct osteocyte infection constitutes a pivotal mechanism in bone infection progression, primarily through *S. aureus*-induced cytokine production by OCs that drives pathological bone loss.

Notably, *S. aureus* employs amoeboid motility to invade and persist within the confined to OLCN (Yu et al., 2020), a process mechanistically linked to PBP3 and/or PBP4 gene expression (da Costa et al., 2018). Suppressing the expression of these two genes may provide novel insights for developing implantable therapeutic materials against bone infections. Bacterial biofilm formation constitutes a critical step in bone infection pathogenesis, as established biofilms impede antibiotic penetration and immune cell infiltration into deep infection sites while resisting mechanical disruption, frequently leading to therapeutic failure (Masters et al., 2019).

The Agr quorum sensing system serves as a key regulator of *S. aureus* biofilm development. Under low cell density conditions, *S. aureus* expresses microbial surface components recognizing adhesive matrix molecules (MSCRAMMs), such as fibronectin-binding proteins A and B (FnBPA and FnBPB), collagen adhesin (Cna), and staphylococcal protein A (SpA), which facilitate bacterial adhesion to abiotic surfaces (Patti et al., 1994). Following adhesion, bacterial proliferation commences. Upon reaching critical population density, the Agr system downregulates MSCRAMMs expression while producing phenol-soluble modulins (PSMs) and virulence factors including α-haemolysin (Hla), Panton-Valentine leukocidin (PVL), and toxic shock syndrome toxin-1 (TSST1), triggering biofilm dispersal (Patti et al., 1994).

**FIGURE 1:**
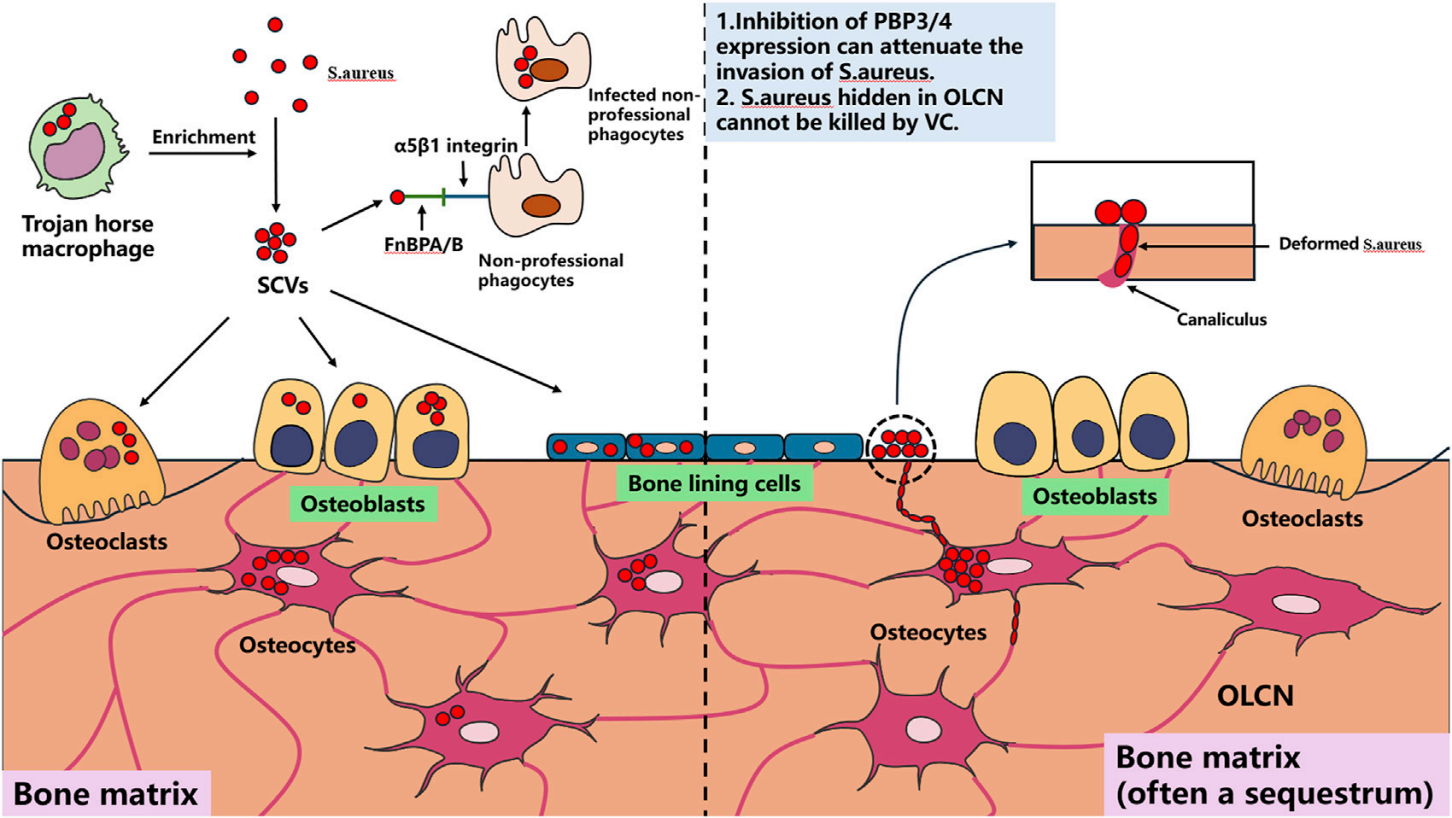
Pathogenesis of *S. aureus* infection bones, self-drawn.

**FIGURE 2:**
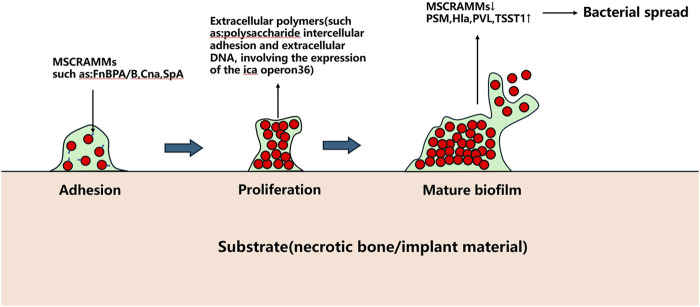
Pathogenesis of *S. aureus* infection bones, self-drawn.

Beyond biofilms, *S. aureus* forms staphylococcal abscess communities (SACs) that contribute to chronic osteomyelitis and peri-implant chronic inflammation progression. SACs persistently acquire iron through iron-scavenging proteins (IdA, IsdB, IsdH) by binding hemoglobin and extracting heme iron for metabolic utilization (Kim et al., 2010). This iron sequestration concurrently compromises local blood supply, exacerbating infection severity (Carek et al., 2001; Kavanagh et al., 2018). Currently, surgical excision remains the sole effective SACs management approach, as these structures demonstrate remarkable resistance to antibiotics and immune attacks. The pathogenic mechanisms of *S. aureus* in bone infections are schematically summarized in Figures 1, 2.

## 3 Advances in antibacterial materials with different matrix types

### 3.1 Metal-based composite implant materials

Clinically utilized metal-based composite implant materials predominantly include: medical-grade stainless steel (316L stainless steel and its low-nickel high-nitrogen modified variants) with superior mechanical properties; wear-resistant cobalt-based alloys (Co-Cr-Mo, Co-Cr-W-Ni alloys); widely applied Ti and its alloys; biodegradable metals (Mg alloys, Zn alloys); and shape-memory alloys (notably nitinol, along with other noble/rare metals primarily employed in dental applications and sensor technologies) (Piñera-Avellaneda et al., 2024).

Ti implants currently represent the most widely used metallic matrix in orthopedic applications. However, challenges persist regarding their osseointegration and interfacial adhesion, making Ti-based implant modification a major focus in materials research with various innovative approaches being proposed (Uicich et al., 2024). Hou et al. developed a nitric oxide (NO)-mediated dual-functional smart Ti implant coating. This system achieves antibacterial efficacy through rapid high-dose NO release in response to infectious microenvironments and near-infrared stimulation, exhibiting antibacterial rates of 97.84% against MRSA and 97.18% against its biofilms. Once infection resolves and physiological conditions normalize, the coating gradually releases low-dose NO to enhance osseointegration (Hou et al., 2025). Hou et al. developed a nitric oxide (NO)-mediated dual-functional smart Ti implant coating. This system achieves antibacterial efficacy through rapid high-dose NO release in response to infectious microenvironments and near-infrared stimulation, exhibiting antibacterial rates of 97.84% against MRSA and 97.18% against its biofilms. Once infection resolves and physiological conditions normalize, the coating gradually releases low-dose NO to enhance osseointegration (Tan et al., 2025). Yu et al. proposed an interfacial functionalization strategy by integrating mesoporous polydopamine nanoparticles (PDA), nitric oxide (NO)-releasing donor sodium nitroprusside (SNP), and osteogenic growth peptide (OGP) onto Ti implants (Ti-PDA@SNP-OGP). Under near-infrared irradiation, this system demonstrated synergistic photothermal and NO-dependent antibacterial effects against MRSA. Through reactive oxygen species (ROS)-mediated oxidative stress induction, it disrupted bacterial membrane integrity, caused intracellular component leakage, and effectively eradicated established MRSA biofilms (Yu et al., 2023). Another study constructed an EGCG/Zn^2+^/MT composite coating on Ti surfaces by loading melatonin (MT), polyphenol (EGCG), and Zn^2+^, achieving 97% and 81% inhibition rates against *E. coil* and *S. aureus* respectively. This coating regulated macrophage (RAW264.7) polarization toward M2 phenotype, induced angiogenesis in human umbilical vein endothelial cells (HUVECs), and promoted osteogenic differentiation of pre-osteoblasts (MC3T3-E1) (Jia et al., 2025). Shen’s team developed Mg/Zn-metal-organic framework (Mg/Zn-MOF74) composite coatings on Ti implants via alkaline thermal treatment. These coatings demonstrated structural stability and pH-responsive degradation in acidic microenvironments generated during bacterial proliferation. The degraded MOF74 coating exhibited potent antibacterial activity against *E. coil* and *S. aureus*. Ti implants with this novel coating showed enhanced early-stage antimicrobial and anti-inflammatory properties *in vivo*, significantly improving peri-implant new bone formation in both non-infected and infected femoral regions (Shen et al., 2019). Similarly, B. Palla-Rubio et al. fabricated hybrid silica-chitosan coatings on Ti implants via sol-gel method. Silicon (Sr), a crucial element for osteogenesis, is continuously released post-implantation, while chitosan exerts bacteriostatic effects (Palla-Rubio et al., 2019). Tomoki Kobatake’s group developed silver (Ag)- HAP coatings on pure Ti implants, demonstrating reduced bacterial burden in femoral bone infections after 14 days, though specific antibacterial mechanisms remain undetermined (Kobatake et al., 2019). Wang et al. engineered a responsive coating on Ti implants that achieves on-demand release of therapeutic hydrogen sulfide (H_2_S) and antibiotics to enhance osseointegration and eradicate infections. This coating sustains low-dose H_2_S release to promote osteogenic differentiation and cellular migration. Bacterial invasion disrupts the dense H_2_S donor layer, triggering rapid antibiotic release for infection prevention. The system exhibits effective antibacterial activity against *E. coil* and *S. aureus* through coordinated H_2_S/antibiotic delivery (Wang et al., 2024). Li et al. employed a layer-by-layer (LBL) assembly technique to sequentially deposit GO and lysozyme (Lys) onto Ti implants, forming an ultrathin film. This film demonstrated effective bactericidal activity against both *S. aureus* and *E. coil* while promoting osteoblast (OB) differentiation, showing promising clinical potential. Other researchers developed hyaluronic acid (HA)/chitosan polyelectrolyte multilayer films on Ti alloy surfaces. The chitosan component prevents bacterial adhesion and biofilm formation through its dense positive charge, while simultaneously serving as a reservoir for antimicrobial triclosan (TRI) (Valverde et al., 2019). Tantalum (Ta) coatings have demonstrated notable antibacterial properties. Zhang’s team modified Ti implants with Ta coatings, where micro-galvanic couples formed between Ta and Ti consume bacterial intracellular protons, reducing ATP synthesis while promoting ROS generation. This process downregulates bacterial virulence genes associated with cellular adhesion, invasion, and viability (Zhang D et al., 2019; Zhang XM et al., 2019). Li’s group engineered a sonosensitive metal-organic framework coating on Ti implants, comprising Cu-TCPP (tetrakis (4-carboxyphenyl)porphyrin) nanosheets and tinidazole-doped outer membrane vesicles (OMVs). Through high-penetration sonodynamic therapy (SDT), Cu-TCPP converts O_2_ to cytotoxic singlet oxygen (^1^O_2_) under normoxic conditions, exacerbating hypoxic microenvironments. Bacterial membrane disruption enhances Cu(I) transporter activity, obstructing the tricarboxylic acid (TCA) cycle and inducing Cu(I) overload, ultimately causing copper-toxicity-mediated bacterial death (Li M et al., 2024; Li S et al., 2024). Ti and Cu can form alloys, where Cu incorporation results in the formation of Cu-rich phases (Ti_2_Cu phase), which is critical for Cu^2+^ release in Ti-Cu alloys. For instance, Geng et al. developed a novel antibacterial osteogenic NSPTICU implant by coating nano-silver particles/poly (lactic-co-glycolic acid) (PLGA) onto Ti6Al4V-Cu surfaces via solvent casting. This system leverages synergistic effects between silver nanoparticles and Cu^2+^, significantly reducing *E. coil* and *S. aureus* colony counts while demonstrating favorable osteogenic properties (Geng et al., 2024). Nelogi et al. constructed iron nanoparticle coatings on Ti implants, which respond to magnetic fields to enhance ROS generation, exhibiting potent antibacterial activity against *E. coil* and *S. aureus* (Nelogi et al., 2024). Nelogi et al. constructed iron nanoparticle coatings on Ti implants, which respond to magnetic fields to enhance ROS generation, exhibiting potent antibacterial activity against *E. coil* and *S. aureus* (Vu et al., 2019). Yang et al. fabricated and evaluated Ti6Al4V-6.5wt%Cu alloy, experimentally confirming its bactericidal action through electrostatic interactions with bacterial positive charges. This alloy accelerates biofilm aging and degradation, reduces C-reactive protein and leukocyte levels, and suppresses local inflammatory responses (Yang et al., 2021). Li et al. constructed a dual drug delivery system (denoted as AH-Sr- silver nanoparticles (AgNPs)) on Ti surfaces capable of independently releasing Sr^2+^ and Ag^+^. This system promoted M2 macrophage polarization for pathogen clearance, partially activated osteoblasts (OBs), and established a pro-healing microenvironment for bone infection resolution (Li et al., 2019). Wang et al. investigated the antibacterial properties of Ti10Cu alloy, demonstrating significantly lower local leukocyte counts and bacterial colony numbers in the Ti10Cu group compared to controls post-implantation, confirming its enhanced antibacterial efficacy (Wang et al., 2019). Zhang’s team integrated vancomycin-loaded hydrogels onto micro-arc oxidized 3D-printed porous Ti6Al4V implants, achieving controlled antibiotic release for precise bacterial proliferation inhibition (Zhang et al., 2024). Shi et al. developed aspirin/amoxicillin-loaded chitosan microparticles combined with PDA-modified Ti implants, experimentally verifying their capacity to suppress *S. aureus* proliferation through antimicrobial action (Shi et al., 2024).

Other modifications have been applied to stainless steel implants. Zhuang et al. developed a Cu-containing 316L stainless steel implant (316L-Cu SS) that demonstrates stable Cu release post-implantation, exhibiting significant antibacterial effects against *E. coil* (95.2% reduction), *S. aureus* (94.8% reduction), and *Staphylococcus* epidermidis (94.1% reduction), demonstrating potential for preventing implant-related infections (IRI) (Zhuang et al., 2020). Qing’s group fabricated Ag-containing coatings on porous stainless steel substrates via 3D printing combined with *in situ* hydrothermal crystallization. The released Ag^+^ effectively inhibits *S. aureus* and *E. coil* growth while maintaining excellent compatibility with bone marrow-derived mesenchymal stem cells (BMSCs) (Qing et al., 2020). Mg-based alloys are considered promising biodegradable orthopedic implants, yet their clinical application is limited by rapid degradation rates and insufficient osteogenic/antibacterial performance. Zhang et al. engineered a dual-layer coating system on Mg alloys comprising an MgF_2_ primer layer and phenolic-amine grafted antimicrobial peptides. This system enhances corrosion resistance, promotes osteogenesis, and achieves over 85% antibacterial rates against both *S. aureus* and *E. coil* (Zhang et al., 2025). Metal-based composite implant materials are systematically summarized in Table 1.

**TABLE 1:** Metal-based composite implant materials.

Base type	Add ingredients	Targeting pathogens	References
Ti implants	NO-mediated dual-function intelligent coating	MRSA	Hou et al. (2025)
Ti implants	Au/MgFe-MMO	Sensitive bacteria	Tan et al. (2025)
Ti implants	PDA, SNP, OGP, NO	MRSA	Yu et al. (2023)
Ti implants	EGCG/Zn^2+^/MT composite coating	*E. coil, S. aureus*	Jia et al. (2025)
Ti implants	Mg/Zn-MOF74	*E. coil, S. aureus*	Shen et al. (2019)
Ti implants	Hybrid silica-chitosan coating	Sensitive bacteria	Palla-Rubio et al. (2019)
Ti implants	Ag, HAP	Sensitive bacteria	Kobatake et al. (2019)
Ti implants	H_2_S, Antibiotics	*E. coil, S. aureus*	Wang et al. (2024)
Ti implants	GO, Lys	*S. aureus, E. coil*	Valverde et al. (2019)
Ti implants	Ta	Sensitive bacteria	Zhang D et al. (2019), Zhang XM et al. (2019)
Ti implants	Cu-TCPP, Tinidazole	Sensitive bacteria	Li M et al. (2024), Li S et al. (2024)
TI6AL4V-Cu alloy	AgNP, PLGA, Cu	*E. coil, S. aureus*	Geng et al. (2024)
Ti implants	Iron nanoparticles	*E. coil, S. aureus*	Nelogi et al. (2024)
Ti6Al4V	ZnO-SiO_2_-Ag_2_O	Sensitive bacteria	Vu et al. (2019)
Ti6Al4V	Cu	Sensitive bacteria	Yang et al. (2021)
Ti implants	AH-Sr-AgNPs	Sensitive bacteria	Li et al. (2019)
Ti10Cu alloy	Cu	Sensitive bacteria	Wang et al. (2019)
316L stainless steel implant	Cu	*E. coil, S. aureus and Staphylococcus epidermis*	Zhuang et al. (2020)
Ti6Al4V	Vancomycin-wrapped hydrogel	Sensitive bacteria	Zhang et al. (2024)
Ti implants	Aspirin/Amocillin-loaded chitosan particles,PDA	*S. aureus*	Shi et al. (2024)
Porous stainless steel metal components	Ag	*S. aureus, E. coil*	Qing et al. (2020)
Mg base alloy	Primer layer/phenominal grafted antimicrobial peptide double coating of MgF2	*S. aureus, E. coil*	Zhang et al. (2025)

Metal-based composite implant materials exhibit several critical limitations: (1) Antimicrobial metal ions (e.g., Ag^+^, Cu^2+^) have been demonstrated to induce cellular protein dysfunction, activate ROS generation, deplete intracellular antioxidants, and cause membrane damage. Systemic accumulation of these ions/particles in lymphatic and circulatory systems may lead to persistent cytotoxicity and increased carcinogenic risks (Teske and Detweiler, 2015). Additionally, nickel-containing alloys pose allergic reaction risks, with cobalt-chromium alloys exhibiting higher allergenic potential. (2) The elastic modulus of metallic materials (e.g., Ti alloys, stainless steel) significantly exceeds that of natural bone, resulting in ineffective load transfer to surrounding bone tissue. This mismatch induces stress shielding effects, triggering bone resorption and osteoporosis, which may ultimately cause implant loosening or fracture. (3) Although inert matrices like commercially pure Ti (cp-Ti) demonstrate mechanical strength, their poor biocompatibility often leads to fibrous encapsulation, compromising long-term osseointegration. (4) Metallic implants generate substantial imaging artifacts in CT/MRI examinations, obscuring critical postoperative evaluations such as bone healing status or tumor recurrence. (5) High manufacturing costs associated with precision forging or 3D printing technologies make metallic implants economically burdensome compared to polymeric/ceramic alternatives.

### 3.2 Bioceramic-based composite implant materials

**FIGURE 3:**
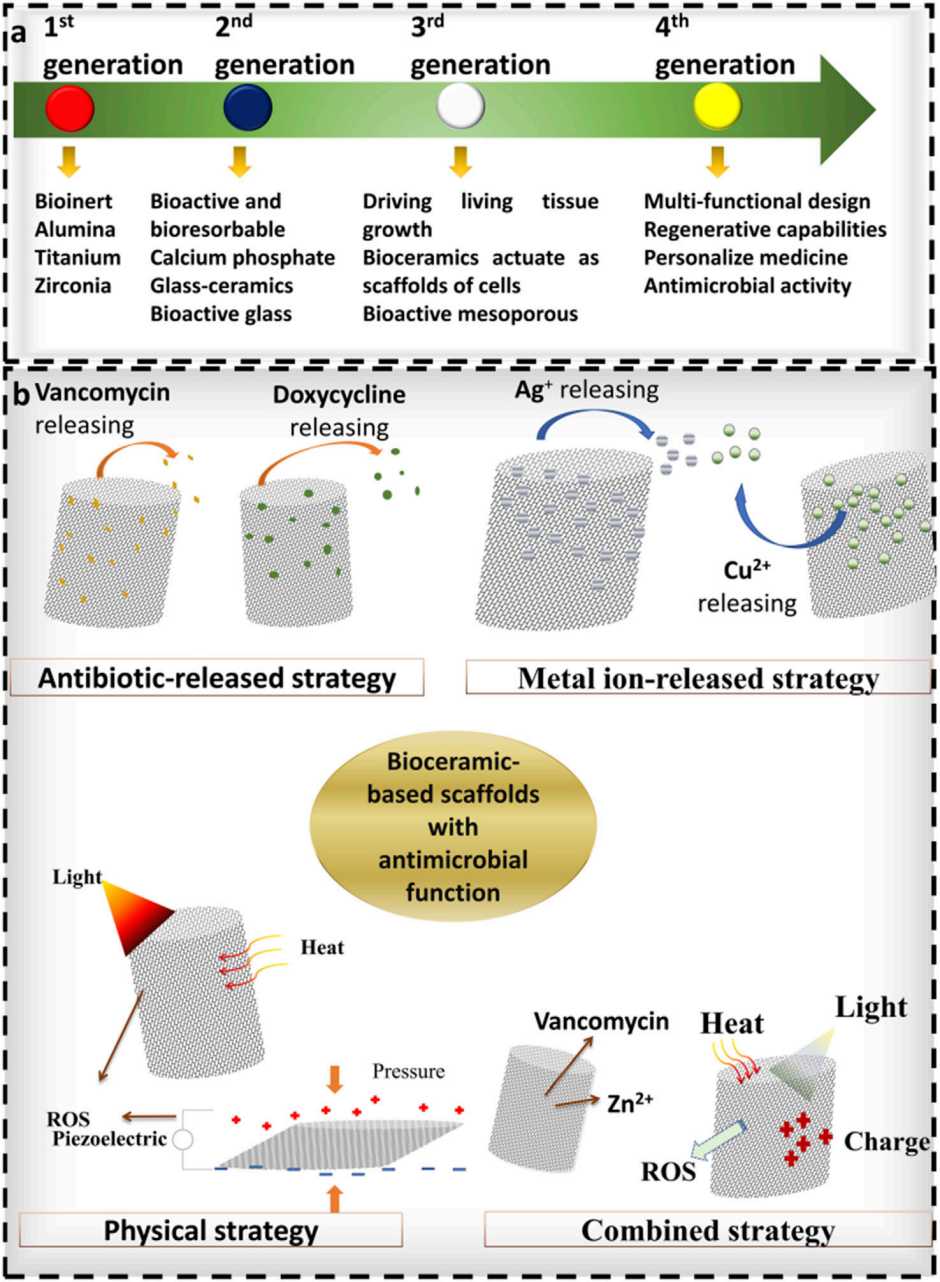
Illustrates the development of bioceramic-based materials, reproduced from (Nguyen et al., 2024) with permission.

Bioceramic matrix materials, including bioactive glass (BG), calcium phosphate, and silicate ceramics, exhibit exceptional osteoconductivity, osteoinductivity, favorable cytocompatibility, low immunogenicity, controllable degradability, multifunctionality, and intrinsic antibacterial properties in certain formulations (Nguyen et al., 2024). Current research focuses on three primary directions: functionalized surface modifications (e.g., coralline alumina), development of multicomponent systems (e.g., HAP/collagen composites), and synergistic integration of antibacterial and therapeutic functions (e.g., nanosilver/hydroxyapatite composites (Bee et al., 2021)). The developmental trajectory of bioceramic-based implant materials is illustrated in Figure 3.

Mahshid Shokri et al. incorporated gallium (Ga) and zinc ions (Zn^2+^) into HAP matrices. The modified material demonstrated >60% antibacterial rates against both *E. coil* and *S. aureus*, while maintaining non-cytotoxicity toward bone marrow-derived mesenchymal stem cells (BMSCs). Co-culture with BMSCs significantly enhanced alkaline phosphatase activity and mineralization capacity, indicating robust osteogenic promotion (Shokri et al., 2022). Yao et al. developed potassium-sodium niobate piezoelectric ceramics (K_0_._5_Na_0_._5_NbO_3_, KNN) and evaluated their biocompatibility and antibacterial efficacy. Results revealed substantial reductions in *S. aureus* colony counts and enhanced proliferation of rat BMSCs (rBMSCs), with mechanisms potentially linked to the surface positive charge of piezoelectric ceramics (Yao T. et al., 2019). Eamon J Sheehy et al. engineered an antibiotic-eluting collagen-HAP scaffold featuring a hierarchical dual-release system. This design enables initial rapid antibiotic release for infection eradication, followed by sustained controlled release to prevent recurrence. The system demonstrates compatibility with gentamicin, vancomycin, and other antibiotics (Sheehy et al., 2025). Zhang et al. synthesized nano-HAP composites containing 3% silver-doped polyurethane (PU) (3% Ag/nHAP/PU), demonstrating controlled Ag^+^ release with effective bactericidal activity. Subsequent studies revealed optimal bone defect repair, moderate scaffold degradation rates, and favorable cytocompatibility in the 3% Ag/nHAP/PU group (Zhang D et al., 2019; Zhang XM et al., 2019). Mg-doped HAP (HAP-Mg) nanofibers, structurally analogous to bone mineral with enhanced biocompatibility and antibacterial performance, were investigated by Ricardo Pascual Alanis-Gómez et al. Their findings revealed dose-dependent inhibitory effects of HAP-Mg nanoparticles against *S. aureus* and *E. coil*. This antibacterial mechanism correlates with Mg^2+^ release, which alters the structural integrity of bacterial cell walls/membranes and induces ROS generation, ultimately leading to bacterial death (Alanis-Gómez et al., 2024). Sathiyavimal et al. fabricated HAP composites using natural waste materials, combined with chitosan and gentamicin sulfate antibiotics (HAP/CS-GA). Experimental results demonstrated potent antibacterial activity against *Klebsiella pneumoniae*, *Pseudomonas aeruginosa*, and *S. aureus* (Sathiyavimal et al., 2024). Hu et al. designed a pH-responsive smart zwitterionic antibacterial coating (PSB/GS coating) deposited on HAP. During initial implantation, the hydrophilic zwitterionic polymer exhibited anti-bacterial adhesion properties, achieving over 90% reduction in protein and bacterial adhesion. Subsequent bacterial proliferation lowered the microenvironmental pH, triggering hydrolysis of acid-sensitive Schiff base bonds to release gentamicin sulfate on-demand, effectively enhancing antibiofilm performance (Hu et al., 2024).

Bian’s team developed a bioactive glass scaffold (BGS) functionalized with MgCuFe-layered double hydroxide (LDH)-derived sulfide nanosheets (BGS/MCFS). This system directly inhibits MRSA energy metabolism, disrupts bacterial membranes, and eliminates peri-implant infections through near-infrared photothermal properties (Bian et al., 2024). As previously described, *S. aureus* residing within OLCN and SACs presents significant therapeutic challenges, with conventional surgical irrigation proving ineffective and contributing to recurrent osteomyelitis. To address this, Jin et al. engineered a borosilicate bioactive glass (BSG) scaffold integrated with 5% ferroferric oxide (Fe_3_O_4_) magnetic nanoparticles. The BSG+5%Fe_3_O_4_ composite enhanced antibacterial immune responses, promoted osteogenic differentiation and mineralization of mesenchymal stem cells (MSCs), and upregulated gene expression of nod-like receptors and TNF pathways in MSCs. Concurrently, it increased osteogenic factor expression (RUNX2, ALP, OCN) while elevating anti-inflammatory genes (TGF-β1, IL-1Ra) and suppressing pro-inflammatory cytokines (IL-6, IL-1β) in macrophages. Under alternating magnetic fields, complete eradication of SACs and bacteria within OLCN was achieved after 42 days of treatment (Jin et al., 2024). Indrakumar developed a drug delivery platform incorporating tannic acid (TA)-silk fibroin (SF) composites into calcium sulfate hemihydrate (CSH). Compared to pure CSH particles, the composite demonstrated a 7.5-fold increase in antioxidant activity and extended antibacterial efficacy by 13 days (Indrakumar et al., 2025). Inna V Fadeeva incorporated boron into β-tricalcium phosphate (β-TCP) bioceramics, revealing that 30% boron-loaded TCP exhibited inhibitory effects against multiple pathogens: 30.9% for *Escherichia coli*, 36.4% for *Enterococcus faecalis*, 37.8% for *Pseudomonas aeruginosa*, 46.8% for *S. aureus*, and 38.8% for *Candida* albicans (Fadeeva et al., 2024). Hayashi et al. developed a carbonate apatite (CAP) matrix with osteoconductivity and biodegradability, partially substituting its surface with Ag_3_PO_4_. This material demonstrated antibacterial activity values exceeding 2 (corresponding to 99% lethality) against MRSA, *S. aureus*, *E. coil*, *Pseudomonas aeruginosa*, *Klebsiella pneumoniae*, *Staphylococcus* epidermidis, VRE, and *Streptococcus* mutans under *in vitro* shaking conditions, exhibiting potent bactericidal efficacy without significant cytotoxicity (Hayashi et al., 2024). Shu et al. fabricated photo-crosslinked methacrylated alginate-co-calcium phosphate cement (PMA-co-PCPC) with antibacterial properties. By employing CuCl_2_ and SrCl_2_ as inhibitors and CaCl_2_ as an activator, the system achieved precisely regulated antimicrobial activity (Shu et al., 2024). Ji et al. leveraged the antibacterial properties of germanium (Ge) and GeO_2_ to augment silicon carbonate (CPS). Results demonstrated that Ge-CPS effectively inhibited *S. aureus* and *E. coil* proliferation. Sustained Ge release during bioceramic degradation ensured prolonged antibacterial activity (Ji et al., 2023). Dina V Deyneko conducted supplementary studies on β-TCP doped with cobalt and nickel. Nickel incorporation exhibited dose-dependent inhibitory effects against *Candida* albicans, *S. aureus*, *E. coil*, and *Enterococcus faecalis*, while showing ambiguous efficacy against *Pseudomonas aeruginosa* (Deyneko et al., 2023). Gaëlle Desante et al. proposed biomimetic calcium phosphate (CaP) coatings encapsulating antibiotic-loaded nanoparticles (gentamicin or bacitracin) to multifunctionalize inert ceramic implants. This approach enhanced both bioactivity and antibacterial efficacy (Desante et al., 2023). Table 2 summarizes bioceramic-based composite implant materials.

**TABLE 2:** Bioceramic-based composite implant materials.

Base type	Add ingredients	Targeting pathogens	References
HAP	Ga and Zn^2+^	*E. coil, S. aureus*	Shokri et al. (2022)
KNN	K, Na	*S. aureus*	Yao et al. (2019b)
Collagen-HAP bracket	Antibiotics	Sensitive bacteria	Sheehy et al. (2025)
Nano HAP	Ag, PU	Sensitive bacteria	Zhang D et al. (2019), Zhang XM et al. (2019)
BGS	Mg, Cu, Fe, LDH	MRSA	Bian et al. (2024)
HAP	Chitosan, Gentamycin sulfate	*Klebsiella pneumoniae,* *Pseudomonas aeruginosa*, *S. aureus*	Sathiyavimal et al. (2024)
BSG	Fe_3_O_4_	*S. aureus*	Jin et al. (2024)
CSH	TA-SF complex, antibacterial drugs	Sensitive bacteria	Indrakumar et al. (2025)
β-TCP	Boron	*E. coil, Enterobacter faecalis, Pseudomonas aeruginosa, S. aureus, Candida albicans*	Fadeeva et al. (2024)
CAP	Ag_3_PO_4_	MRSA, *S. aureus, E. coil,* *Pseudomonas aeruginosa*, *Klebsiella pneumoniae, Staphylococcus epidermis*, VRE and *Streptococcus mutans*	Hayashi et al. (2024)
CaP cement	Methacrylate alginate	Sensitive bacteria	Shu et al. (2024)
HAP	Mg	*S. aureus, E. coil*	(Alanis-Gómez et al., 2024)
HAP	PSB/GS coating, gentamicin sulfate	Sensitive bacteria	Hu et al. (2024)
CPS	Ge, GeO_2_	*S. aureus, E. coil*	Ji et al. (2023)
β-TCP	Cobalt, Nickel	*Candida albicans, S. aureus, E. coil,* *Enterobacter* faecalis	Deyneko et al. (2023)
Inert ceramic implant	CaP, Antibiotic nanoparticles	Sensitive bacteria	Desante et al. (2023)

The defects of bioceramic-based composite implant materials are mainly as follows: (1) Fragile. Although their hardness is high, bioceramic-based materials are prone to fracture under high stress conditions (such as the fragmentation rate is as high as 15% during inlay repair), and this risk will further increase during large-scale repair applications; (2) The elastic modulus is mismatched, such as zirconia (elastic modulus about 200 GPa) is much higher than that of human bone (7–30 GPa), which may lead to stress shielding effect, and long-term implantation triggers bone resorption; (3) Poor fatigue resistance, and under repeated stress environments (such as artificial joints), ceramic materials are prone to failure due to microcrack propagation, and need to be improved through toughening technology (such as nanocomposite). (4) The rate of degradation *in vivo* may not match the rate of bone growth, resulting in the risk of repair failure; (5) Risk of biocompatibility. A few patients may have allergies or local inflammatory reactions to ceramic components (such as certain phosphates or metal ions doping) (the incidence rate is about 1%–3%), and there is also the possibility of bioceramic-based materials that cannot be integrated with human bones, and long-term stress weakness may occur. (6) The production cost of bioceramic-based materials is relatively high and processing is difficult. These limit the widespread application of bioceramic-based materials in clinical practice. At present, various ways have been improved to address these problems of bioceramic-based implant materials, such as making composite materials by doping nanomaterials for increasing toughness treatment. Some researchers have also combined Mg alloys or polymers with bioceramic materials to balance the relationship between material strength and degradation rate.

### 3.3 Polymer-based composite implant materials

Polymer-based implants represent another major category for bone infection defect repair, including classical materials such as PMMA (Ghavimi et al., 2019), polyetheretherketone (PEEK) (Zheng et al., 2024), polylactic acid (PLA), PU (Hu et al., 2014), and polyethylene. These materials generally exhibit favorable biocompatibility. Certain formulations, notably PEEK, possess elastic moduli comparable to human bone, thereby mitigating stress shielding-induced bone resorption. PEEK is regarded as the most viable alternative to Ti. However, these polymers inherently lack antibacterial activity. Functional enhancements can be achieved through chemical modifications or composite material incorporation to improve mechanical properties (e.g., hardness, fatigue resistance), osteoconductivity, and antimicrobial performance.

Similar to Ti-based implants, surface modification of PEEK remains a key focus in polymer-based implant research. Zhao et al. loaded L-arginine onto sulfonated PEEK (SPEEK). Under infectious conditions, inducible nitric oxide synthase (iNOS) catalyzes L-arginine to generate NO and ROS, exerting bactericidal effects. Additionally, this system induces macrophage polarization toward M1 and M2 phenotypes, providing indirect antibacterial action (Zhao et al., 2024). Chen et al. developed a hydrogel coating (GPL) composed of gelatin methacryloyl (GelMA), methacrylamide-modified ε-polylysine (ε-PLMA), and laponite, crosslinked onto PEEK via UV irradiation. This surface modification enhances osteogenic capacity while maintaining antibacterial activity against *S. aureus* and *E. coil* until complete hydrogel hydrolysis (Chen et al., 2023). Han et al. applied the natural antimicrobial agent paclitaxel as a coating on fused filament fabrication (FFF) 3D-printed PEEK. At a paclitaxel concentration of 10 mg/mL, the coating exhibited contact-killing effects that inhibited biofilm development (Han et al., 2024). Wei et al. modified SPEEK surfaces with Ag^+^-incorporated GO films. The modified material inhibited proliferation of *S. aureus*, *E. coil*, *Klebsiella pneumoniae*, *Candida albicans*, and *Pseudomonas aeruginosa*, while reducing bacterial adhesion and biofilm formation. GO incorporation enhanced Ag^+^ release, optimizing bactericidal efficacy (Wei et al., 2024). Sun et al. fabricated gentamicin-loaded chitosan coatings on porous PEEK fixation plates, demonstrating superior antibacterial activity against *S. aureus* and *E. coil* (Sun et al., 2025). Notably, Liu et al. innovatively immobilized siRNA onto PEEK surfaces using antimicrobial polyphenol tannic acid (pTAN). pTAN inhibits bacterial adhesion by suppressing glycosyltransferase activity, increases membrane permeability, and ultimately induces cytoplasmic leakage leading to bacterial death (Liu et al., 2024). Şakir Altınsoy et al. incorporated AgNPs and copper nanoparticles (CuNPs) onto PEEK surfaces for evaluation. Results demonstrated that AgNPs exhibited superior antioxidant properties compared to CuNPs, significantly enhancing PEEK’s surface activity and antibacterial performance (Altınsoy et al., 2024).

Carbon fiber-reinforced polyetheretherketone (CFRPEEK), a modified PEEK variant, is considered a promising biomedical implant material. However, its inertness and insufficient antibacterial activity limit clinical utility. Wang et al. modified CFRPEEK via Ti plasma immersion ion implantation and hybridization with PDA@ZnO-EDN1 nanoparticles. The modified CFRPEEK effectively inhibited *S. aureus* and *E. coil* adhesion/aggregation, significantly increasing bacterial membrane permeability. Subsequent leakage of intracellular components (e.g., proteins and adenosine triphosphate, ATP) induced bacterial death (Wang J et al., 2023; Wang X et al., 2023). Diabetic patients frequently suffer from comorbid bone infections, where the diabetic infection microenvironment (DIME) – characterized by hyperglycemia and peri-implant pathogenic infections–often leads to bone repair failure. To address this, He et al. engineered a glucose-responsive orthopedic implant comprising PEEK, a copper-chelated metal-polyphenol network (hauberk coating), and glucose oxidase (GOx). Within DIME, GOx continuously consumes glucose to generate H_2_O_2_, while the hauberk coating releases Cu to catalyze H_2_O_2_ into highly bactericidal hydroxyl radicals (•OH). Subsequent photo-enhanced chemodynamic therapy eradicates pathogens, offering a novel strategy for refractory diabetic Bone infection (He et al., 2023). Li et al. developed a dual-functional surface modification strategy by achieving sustained release of chondroitin sulfate (CS) and levofloxacin (LVFX) from a bio-inspired PDA coating on PEEK. LVFX release provided localized bactericidal effects and promoted bone defect repair (Li M et al., 2024; Li S et al., 2024). Gao et al. immobilized the antimicrobial peptide HHC36 onto the three-dimensional porous structure of SPEEK and conducted characterization. Experiments demonstrated sustained HHC36 release over 10 days from SPEEK, reducing planktonic bacterial survival rates, suppressing peri-sample bacterial growth, and inhibiting surface biofilm formation (Gao et al., 2023). Enhancements have also been designed for other materials.

Fan et al. incorporated BSG into low-dose gentamicin sulfate-loaded PMMA cement to create an intelligent antibiotic release system. This strategy synergistically eradicated bacteria and promoted osseointegration by disrupting bacterial cell wall/membrane integrity, inhibiting ATP synthesis via respiratory chain and glycogen metabolism interference, and elevating ROS levels through depletion of antioxidant components (peroxisomes and carotenoids), achieving efficient bacterial killing and biofilm eradication (Fan et al., 2025). Ankita Negi et al. fixed citric acid-conjugated chitosan onto oxygen plasma-etched 3D-printed PLA scaffolds via EDC-NHS coupling. Compared to unmodified oxygen plasma-etched PLA scaffolds, the surface-modified scaffolds exhibited superior antibacterial activity against *S. aureus* and *E. coil* (Negi et al., 2024). Mohammad Reza Shafiei et al. incorporated rifampicin-loaded mesoporous silica into acrylic bone cement to address antimicrobial resistance in coagulase-negative staphylococcal biofilms. Results demonstrated that the modified cement enhanced mechanical strength and optimized drug release kinetics, achieving bactericidal efficacy while minimizing resistance development (Shafiei et al., 2025). Olga Bakina et al. developed novel PMMA-based HAP/ZnFe_2_O_4_/ZnO composites using ZnFe_2_O_4_/ZnO heterostructures as antibacterial agents. The material exhibited >99% inhibition against *Pseudomonas aeruginosa*, *S. aureus*, and MRSA, with 100% efficacy against *Candida* albicans. This activity likely stems from ZnFe_2_O_4_/ZnO-induced oxidative damage to cell membranes, proteins, and DNA, coupled with electrostatic gradient disruption leading to bacterial inactivation (Bakina et al., 2023). Ultra-high molecular weight polyethylene (UHMWPE) represents an emerging matrix material. Mehmet D Asik et al. blended submicron gentamicin sulfate particles into UHMWPE, demonstrating significantly higher and sustained antibiotic release rates alongside improved mechanical properties, suggesting potential for IRI management (Asik et al., 2025). Li et al. fabricated a multifunctional gelatin methacryloyl/methacrylated dopamine adhesive hydrogel coating containing polydopamine-protected 2D black phosphorus (BP) nanoparticles via photocrosslinking, modifying polyarylethernitrileketone (PPENK) implant surfaces. The system enables photothermal-controlled drug delivery (e.g., doxorubicin hydrochloride) and photodynamic bacterial eradication. pBP’s photothermal effects regulate drug release via electrostatic interactions while generating ROS for bactericidal action, with ROS scavenging capabilities preventing normal cell apoptosis (Li J et al., 2023; Li Y et al., 2023). Table 3 summarizes Polymer-based composite implant materials.

**TABLE 3:** Polymer-based composite implant materials.

Base type	Add ingredients	Targeting pathogens	References
SPEEK	L-arginine	Sensitive bacteria	Zhao et al. (2024)
PEEK	GelMA,ε-PLMA,GPL	*S. aureus, E. coil*	Chen et al. (2023)
PEEK	Taxol	Sensitive bacteria	Han et al. (2024)
SPEEK	Ag^+^,GO	*S. aureus*,* E. coil*, *Klebsiella pneumoniae*, *Candida* and *Pseudomonas aeruginosa*	Wei et al. (2024)
PEEK	Gentamicin, chitosan coating	*S. aureus, E. coil*	Sun et al. (2025)
PEEK	siRNA	Sensitive bacteria	Liu et al. (2024)
PEEK	AgNP, CuNP	Sensitive bacteria	(Altınsoy et al., 2024)
CFRPEEK	PDA@ZnO-EDN1, Ti ion	*S. aureus*, *E. coil*	Wang J et al. (2023), Wang X et al. (2023)
PEEK	Hauberk coating, GOx	Sensitive bacteria	He et al. (2023)
SPEEK	HHC36	Sensitive bacteria	Gao et al. (2023)
PMMA	BSG, Gentamycin sulfate	Sensitive bacteria	Fan et al. (2025)
PLA Bracket	Chitosan	*S. aureus*, E. coil	Negi et al. (2024)
Acrylic bone cement	Rifampin, mesoporous silica	Sensitive bacteria	Shafiei et al. (2025)
PMMA	HAP, ZnFe_2_O_4_, ZnO	*Pseudomonas aeruginosa*, *S. aureus*, MRSA, *Candida albicans*	Bakina et al. (2023)
UHMWPE	Submicron gentamicin sulfate granules	Sensitive bacteria	Asik et al. (2025)
PPENK	Gelatin methacrylate/dopamine methacrylate adhesive hydrogel coating	Sensitive bacteria	Li J et al. (2023), Li Y et al. (2023)

Polymer-based composite implants are not without limitations. These materials exhibit limited load-bearing capacity due to elastic moduli that, while similar to skin and bone, remain significantly lower than those of metallic and ceramic-based materials, posing fracture risks in weight-bearing applications. Long-term use of UHMWPE may generate wear particles through oxidative degradation, potentially leading to osteolysis, prosthetic loosening, and revision surgeries. Biocompatibility challenges persist, including insufficient osseointegration of PEEK implants and non-specific protein adsorption that activates macrophages to release pro-inflammatory factors, exacerbating postoperative inflammation. Degradable polymers like PLA and PLGA face mismatched degradation-osteogenesis rates, while localized accumulation of degradation products may exacerbate bacterial-induced acidic microenvironments, potentially inhibiting OB activity. Additionally, complex manufacturing processes and high R&D costs hinder clinical translation, exemplified by the substantially higher development costs of fourth-generation ceramic-reinforced PEEK compared to metallic alternatives. Current strategies focus on nanocomposite integration (e.g., CFRPEEK) to enhance mechanical strength while preserving PEEK’s inherent advantages, surface functionalization to develop smart responsive materials, and 3D printing-based.

### 3.4 Other composite materials

Beyond the aforementioned three matrix types, other composite materials have achieved notable research progress. Addressing PLA’s limitations, Hyun Lee et al. developed PLA/Mg composites combining Mg with PLA. The composite retains PLA’s biodegradability while incorporating Mg’s intrinsic antibacterial properties and NIR-induced photothermal effects, synergistically minimizing IRI (Lee et al., 2023). Cheng et al. uniformly distributed silver nanoparticles within PLA/gelatin composite fibers via co-electrospinning. The composite demonstrated >97% antibacterial rates against *Candida* albicans, *S. aureus*, *E. coil*, and *Pseudomonas aeruginosa*, alongside enhanced adhesion and proliferation of MSCs and human umbilical vein endothelial cells (HUVECs), indicating robust osteogenic activity (Cheng et al., 2020). Liu et al. constructed PDA self-polymerized films on PLA/HAP nanofibers, followed by stable silver nanoparticle coatings for controlled release. The composite fibers exhibited excellent physiological stability, 100% antibacterial efficacy against *S. aureus* and *E. coil*, and favorable osteoblast compatibility (Liu et al., 2020). Shi et al. engineered an infection-responsive guided tissue regeneration (GTR)/guided bone regeneration (GBR) membrane based on electrospun nanofibers. Metronidazole (MNA) was esterified and covalently linked via ester bonds to modified polycaprolactone (PCL) nanofiber mats. Under infection, cholesterol esterase (CE) secreted by macrophages hydrolyzes these ester bonds to release MNA, with CE concentrations correlating to bacterial infection severity, enabling infection-responsive drug release (Shi et al., 2019). Tang et al. developed nano zinc magnesium silicate (nZMS)/PK bioactive composites (nZPC). Increasing nZMS content significantly enhanced MC3T3-E1 cell adhesion, proliferation, and differentiation on nZPC. Maximum inhibition against *E. coil* was achieved at 50% nZMS loading (Tang et al., 2019).

Huang et al. designed a nanoreactor integrating piezoelectric barium titanate with PDA and Cu. Ultrasound activation generates piezoelectric hot carriers transferred by PDA to Cu, promoting ROS generation and Cu^2+^/Cu^+^ interconversion. This facilitates hydroxyl radical (•OH) production via Cu^+^-catalyzed chemodynamic reactions. Elevated ROS levels induce bacterial membrane disruption, DNA damage, and Cu overload-mediated TCA cycle inhibition, culminating in copper-toxicity-like bacterial death (Huang et al., 2024). Ujjayan Majumdar designed a Ti6Al4V disk coated with HAP, featuring a dual drug delivery system with curcumin in the lower layer and vitamin C in the upper layer. Curcumin and vitamin C releases demonstrated 94% and 98.6% antibacterial efficiency against *S. aureus*, respectively. Bacteria exposed to these agents exhibited either expanded membranes or compromised cellular integrity, confirming bactericidal efficacy of the implant design (Majumdar and Bose, 2024). Xie et al. developed a multifunctional collagen composite scaffold (CUR-GO-COL) by freeze-drying crosslinked curcumin-loaded GO and collagen. The composite achieved sustained curcumin release *in vitro*, reducing *S. aureus* and *E. coil* colony counts. This activity likely stems from GO’s edge-induced mechanical membrane damage and curcumin’s inherent antibacterial properties, synergistically compromising bacterial structural integrity and viability (Xie et al., 2024). Weronika Bodylska et al. combined biocompatible HAP with Ti-based metal-organic framework MIL-125(Ti)-NH_2_, impregnated with gentamicin to form a novel composite. Gentamicin integration significantly enhanced bactericidal effects against *S. aureus* and *Pseudomonas aeruginosa*, highlighting metal-organic framework (MOF)-based composites as emerging research frontiers (Bodylska et al., 2024). Mansi Uday Joshi et al. engineered a pH-responsive alginate composite delivery system for tobramycin. Acidic infection microenvironments triggered responsive drug release, achieving >84% inhibition against *S. aureus* within 30 min (Joshi et al., 2023).

Researchers have also fabricated antibacterial materials by combining alginate with Zn, lactate, HAP, and other components. These composites exhibit excellent antibacterial performance and moderate osteogenic promotion capabilities (Suo et al., 2020; Beheshtizadeh et al., 2023; Tanadchangsaeng et al., 2024). Li et al. developed a hydrogel delivery vehicle loaded with DNase I and vancomycin. After implantation in rats, the system initially co-released DNase I and vancomycin, followed by sustained vancomycin release for up to 14 days, thus effectively eliminating MRSA infections, preventing biofilm formation, and providing a sterile microenvironment conducive to healing fracture-associated infections (Li J et al., 2023; Li Y et al., 2023). Wang et al. incorporated HAP microspheres into GelMA hydrogels via photocrosslinking to create multifunctional injectable hydrogels. These materials demonstrated enhanced flexural strength, cell adhesion sites, and antibacterial functionality through metal ion incorporation. The Ag-incorporated HAP/GelMA hydrogel showed significant inhibition against *Escherichia coli* but weaker efficacy against *S. aureus*, likely attributable to the thicker cell walls and inherent antimicrobial resistance of Gram-positive bacteria (Wang J et al., 2023; Wang X et al., 2023). Abdullah Mohammed et al. proposed a novel PLA/calcium peroxide (PLA/CPO) composite filament for 3D-printed scaffolds. Calcium and oxygen released from the material exhibited antibacterial activity against Gram-positive bacteria, which may be attributed to residual hydrogen peroxide generated during CPO decomposition (Mohammed et al., 2023).

## 4 Degradation effects and impacts of antimicrobial implant materials

Similar to materials utilized in other fields, antimicrobial implants cannot persist indefinitely in a pristine state post-implantation, as degradation ensures following introduction into the biological environment. The degradation effects refer to the progressive deterioration of antimicrobial efficacy and other material properties under physicochemical influences *in vivo*. These degradation manifestations primarily include: Uncontrolled release of antimicrobial agents, Biotoxicity of degradation byproducts, Decline/loss of mechanical strength and Delamination and functional loss of surface structures. Existing experimental evidence confirms multiple adverse impacts of degradation effects on host tissues. The uncontrolled release of antimicrobial agents may elevate local concentrations to cytotoxic levels, thereby compromising osteogenic activities (Feng et al., 2018). Current antimicrobial implants often incorporate AgNPs. Prolonged bacterial exposure to sublethal AgNP concentrations may activate the bacterial SOS repair system (Janion, 2008), potentially leading to enhanced biofilm formation and genetic mutations. Rapid degradation of Mg alloys can elevate local pH levels, creating alkaline microenvironments that induce tissue damage while prematurely compromising antimicrobial efficacy (Jin et al., 2014). Degradation-released metal ions (e.g., Ag^+^, Ni^2+^) may accumulate in organs such as the liver and kidneys, causing systemic toxicity. Circulating nickel ions may trigger allergic responses. Mechanical strength deterioration risks stress shielding effects or implant fracture, which could catastrophically result in secondary fractures and osseointegration failure for bone defect patients. Under stress conditions, carbon fiber-reinforced composites subjected to prolonged stress release particulate debris, exacerbating localized inflammatory responses (Kang et al., 2012). Furthermore, antimicrobial coatings represent a critical surface modification strategy for diverse implant materials. However, coating delamination can diminish or abolish antimicrobial efficacy. For instance, PDA-mediated Mg^2+^/ceria oxide coatings may prematurely exhaust antibacterial payloads in acidic infectious microenvironments due to pH-responsive release mechanism failure (Aurore et al., 2018). Degradation of photothermal-responsive materials (e.g., Au@ZIF-8) diminishes photocatalytic ROS generation capacity (Ortiz-Benítez et al., 2019). Residual bacteria not fully eradicated by degraded photodynamic coatings may induce chronic infections (Yao T. et al., 2019), complicating subsequent therapeutic interventions.

## 5 Summary and perspectives

Although bone infection remains a devastating skeletal disease, novel intelligent materials continue to emerge. This review systematically categorizes antimicrobial implant materials for bone infection defect repair (2019–2025, with emphasis on 2024–2025) based on matrix types, encompassing both newly developed materials and enhanced traditional systems. Most materials exhibit multifunctionality, integrating antibacterial efficacy with osteogenic promotion and immunomodulatory capabilities. We comprehensively discuss advances in: Antimicrobial metal-based composite implants, antimicrobial bioceramic-based composite implants, antimicrobial polymer-based composite implants and other specialized antimicrobial composite systems. Concurrent attention is given to material degradation effects. Despite inherent application-specific limitations, encouraging progress is evident. Emerging research prioritizes: Functional integration: Transition from single antibacterial functions to synergistic antibacterial-osteogenic systems; Intelligent responsiveness: pH-/photothermal-responsive materials; Bioinspired precision: Bacteria-responsive antimicrobial release mimicking physiological immune environments. These developments expand therapeutic options for bone infection management.

The future development of various materials mainly includes the following directions. For metal-based composite implant materials, the first direction is the potential transition from mechanical adaptation to controllable degradation. The developed Ti-Ta and Ti-niobium (Ti-Nb) low-modulus alloys and gradient porous structures (e.g., 3D-printed porousTi) (Sun et al., 2023) are designed to address the mismatch between elastic modulus and bone. Additionally, regulating the degradation rate by adding rare metal elements to Mg alloys and Zn alloys may enable dynamic matching between degradation rates and bone regeneration in the future, or accelerate the release of antibacterial metal ions under infectious microenvironments. Finally, functionalized surface modifications can enhance osseointegration capacity, promote the role of local immune cells, and reduce the likelihood of local allergic reactions (Zhang et al., 2023). For bioceramic-based composite implant materials, the focus lies in nanocomposite reinforcement, degradation regulation, intelligent responsiveness (pH- or enzyme-responsive ceramics), and functional structural design (4D-printed bioceramic materials, shape-memory ceramics (zirconia-based bioceramic materials)), aiming to transform bioceramics from inert materials into intelligent responsive materials. The development of polymer-based composite implant materials also follows the directions of nanoscale engineering, intelligent responsiveness, and controlled degradation rates. Meanwhile, most composite materials are no longer simple combinations of components but emphasize synergistic interactions, gradient composites, and biomimetic multimodal development. Materials are no longer limited to antibacterial properties but strive to design multifunctional implants (Zhang et al., 2023). While retaining antibacterial capabilities, electrical stimulation can be applied to promote osteogenesis at bone defect sites. Furthermore, advancements in novel technologies such as 4D printing technology (a printing technology capable of manufacturing “dynamic” objects responsive to external stimuli), 3D bioprinting technology, and *in situ* manufacturing technology are shifting implant materials from standardized mass production toward personalized designs. For example, some studies integrate 4D printing with smart materials to create antibacterial implants that adapt to anatomical structures (Zhou et al., 2023). The integration of artificial intelligence (AI) enables material production machines to intelligently learn parameters, optimize cost and quality issues during manufacturing, and reduce the usage costs of antimicrobial materials. Current research on antibacterial materials involves interdisciplinary collaborations. By combining single-cell sequencing with bone infection models, researchers aim to elucidate the regulatory mechanisms of OB competitive adhesion on material surface topologies (e.g., nanopillar arrays), guiding the design of multifunctional antibacterial surfaces.

In summary, the development of novel antibacterial materials is still ongoing. We earnestly hope that future antibacterial implant materials for bone defects can maintain implantation performance and localized antibacterial efficacy over the long term, ultimately achieving permanent implantation.

## References

[B1] Aktuglu K. Erol K. Vahabi A. (2019). Ilizarov bone transport and treatment of critical-sized tibial bone defects: a narrative review. J. Orthop. Traumatol. 20, 22. 10.1186/s10195-019-0527-1 30993461 PMC6468024

[B2] Alanis-Gómez R. P. Hernández-Rosas F. Olivares-Hernández J. D. Rivera-Muñoz E. M. Zapatero-Gutiérrez A. Méndez-Lozano N. (2024). Magnesium-doped hydroxyapatite nanofibers for medicine applications: characterization, antimicrobial activity, and cytotoxicity study. Int. J. Mol. Sci. 25 (22), 12418. 10.3390/ijms252212418 39596483 PMC11594928

[B3] Aljohani S. Layqah L. Masuadi E. Al Alwan B. Baharoon W. Gramish J. (2020). Occurrence of vancomycin MIC creep in methicillin resistant isolates in Saudi Arabia. J. Infect. Public Health 13, 1576–1579. 10.1016/j.jiph.2020.07.008 32859551

[B4] Al Thaher Y. Yang L. Jones S. A. Perni S. Prokopovich P. (2018). LbL-assembled gentamicin delivery system for PMMA bone cements to prolong antimicrobial activity. PLoS One 13, e0207753. 10.1371/journal.pone.0207753 30543660 PMC6292632

[B5] Altınsoy Ş. Kızılbey K. İlim H. B. (2024). Green synthesis of Ag and Cu nanoparticles using E. telmateia ehrh extract: coating, characterization, and bioactivity on PEEK polymer substrates. Mater. (Basel) 17 (22), 5501. 10.3390/ma17225501 PMC11595242 39597325

[B6] Asik M. D. Walsh-Rock E. Inverardi N. Nepple C. Zhao T. Sekar A. (2025). Enhanced antibiotic release and mechanical strength in UHMWPE antibiotic blends: the role of submicron gentamicin sulfate particles. J. Bone Jt. Surg. Am. 107 (6), 586–593. 10.2106/jbjs.24.00689 39847614

[B7] Aurore V. Caldana F. Blanchard M. Kharoubi Hess S. Lannes N. Mantel P. Y. (2018). Silver-nanoparticles increase bactericidal activity and radical oxygen responses against bacterial pathogens in human osteoclasts. Nanomedicine 14, 601–607. 10.1016/j.nano.2017.11.006 29155361

[B8] Bakina O. Svarovskaya N. Ivanova L. Glazkova E. Rodkevich N. Evstigneev V. (2023). New PMMA-based Hydroxyapatite/ZnFe(2)O(4)/ZnO composite with antibacterial performance and low toxicity. Biomimetics (Basel) 8 (6), 488. 10.3390/biomimetics8060488 37887619 PMC10604293

[B9] Bee S. L. Bustami Y. Ul-Hamid A. Lim K. Abdul Hamid Z. A. (2021). Synthesis of silver nanoparticle-decorated hydroxyapatite nanocomposite with combined bioactivity and antibacterial properties. J. Mater Sci. Mater Med. 32, 106. 10.1007/s10856-021-06590-y 34426879 PMC8382650

[B10] Beheshtizadeh N. Farzin A. Rezvantalab S. Pazhouhnia Z. Lotfibakhshaiesh N. Ai J. (2023). 3D printing of complicated GelMA-coated Alginate/tri-Calcium silicate scaffold for accelerated bone regeneration. Int. J. Biol. Macromol. 229, 636–653. 10.1016/j.ijbiomac.2022.12.267 36586652

[B11] Bian Y. Hu T. Zhao K. Cai X. Li M. Tan C. (2024). A LDH-derived metal sulfide nanosheet-functionalized bioactive glass scaffold for vascularized osteogenesis and periprosthetic infection prevention/treatment. Adv. Sci. (Weinh) 11 (39), e2403009. 10.1002/advs.202403009 39159063 PMC11497026

[B12] Bodylska W. Junka A. Brożyna M. Bartmański M. Gadzała-Kopciuch R. Jaromin A. (2024). New biocompatible Ti-MOF@hydroxyapatite composite boosted with gentamicin for postoperative infection control. ACS Biomater. Sci. Eng. 10 (12), 7555–7565. 10.1021/acsbiomaterials.4c01230 39592942

[B13] Carek P. J. Dickerson L. M. Sack J. L. (2001). Diagnosis and management of osteomyelitis. Am. Fam. Physician 63, 2413–2420. 11430456

[B14] Chen Y. Chen Y. Han T. Xie Z. Yang Y. Chen S. (2023). Enhanced osteogenic and antibacterial properties of polyetheretherketone by ultraviolet-initiated grafting polymerization of a gelatin methacryloyl/epsilon-poly-L-lysine/laponite hydrogel coating. J. Biomed. Mater Res. A 111 (11), 1808–1821. 10.1002/jbm.a.37589 37548424

[B15] Cheng X. Wei Q. Ma Y. Shi R. Chen T. Wang Y. (2020). Antibacterial and osteoinductive biomacromolecules composite electrospun fiber. Int. J. Biol. Macromol. 143, 958–967. 10.1016/j.ijbiomac.2019.09.156 31739052

[B16] da Costa T. M. de Oliveira C. R. Chambers H. F. Chatterjee S. S. (2018). PBP4: a new perspective on *Staphylococcus aureus* β-Lactam resistance. Microorganisms 6, 57. 10.3390/microorganisms6030057 29932109 PMC6164785

[B17] de Mesy Bentley K. L. Trombetta R. Nishitani K. Bello-Irizarry S. N. Ninomiya M. Zhang L. (2017). Evidence of Staphylococcus aureus deformation, proliferation, and migration in canaliculi of live cortical bone in murine models of osteomyelitis. J. Bone Min. Res. 32, 985–990. 10.1002/jbmr.3055 PMC5413415 27933662

[B18] Desante G. Pudełko I. Krok-Borkowicz M. Pamuła E. Jacobs P. Kazek-Kęsik A. (2023). Surface multifunctionalization of inert ceramic implants by calcium phosphate biomimetic coating doped with nanoparticles encapsulating antibiotics. ACS Appl. Mater Interfaces 15 (17), 21699–21718. 10.1021/acsami.3c03884 37083334

[B19] Deyneko D. V. Lebedev V. N. Barbaro K. Titkov V. V. Lazoryak B. I. Fadeeva I. V. (2023). Antimicrobial and cell-friendly properties of cobalt and nickel-doped tricalcium phosphate ceramics. Biomimetics (Basel) 9 (1), 14. 10.3390/biomimetics9010014 38248588 PMC10813436

[B20] Ding J. Xiao H. Chen X. (2022). Advanced biosafety materials for prevention and theranostics of biosafety issues. Biosaf. Health 4, 59–60. 10.1016/j.bsheal.2022.03.011 35313507 PMC8926432

[B21] Driscoll J. A. Lubbe R. Jakus A. E. Chang K. Haleem M. Yun C. (2020). 3D-Printed ceramic-demineralized bone matrix hyperelastic bone composite scaffolds for spinal fusion. Tissue Eng. Part A 26, 157–166. 10.1089/ten.tea.2019.0166 31469055 PMC7044791

[B22] Dudareva M. Hotchen A. J. Ferguson J. Hodgson S. Scarborough M. Atkins B. L. (2019). The microbiology of chronic osteomyelitis: changes over ten years. J. Infect. 79, 189–198. 10.1016/j.jinf.2019.07.006 31319142

[B23] Dziewanowska K. Patti J. M. Deobald C. F. Bayles K. W. Trumble W. R. Bohach G. A. (1999). Fibronectin binding protein and host cell tyrosine kinase are required for internalization of *Staphylococcus aureus* by epithelial cells. Infect. Immun. 67, 4673–4678. 10.1128/iai.67.9.4673-4678.1999 10456915 PMC96793

[B24] Edwards A. M. Potts J. R. Josefsson E. Massey R. C. (2010). *Staphylococcus aureus* host cell invasion and virulence in sepsis is facilitated by the multiple repeats within FnBPA. PLoS Pathog. 6, e1000964. 10.1371/journal.ppat.1000964 20585570 PMC2891841

[B25] Fadeeva I. V. Barbaro K. Altigeri A. Forysenkova A. A. Gafurov M. R. Mamin G. V. (2024). Exploring borate-modified calcium phosphate ceramics: antimicrobial potential and cytocompatibility assessment. Nanomater. (Basel) 14 (6), 495. 10.3390/nano14060495 PMC10973998 38535643

[B26] Fan M. Ren Y. Zhu Y. Zhang H. Liu C. Lv H. (2025). Borosilicate bioactive glass synergizing low-dose antibiotic loaded implants to combat bacteria through ATP disruption and oxidative stress to sequentially achieve osseointegration. Bioact. Mater 44, 184–204. 10.1016/j.bioactmat.2024.10.009 39502840 PMC11535878

[B27] Feng Y. Zhu S. Wang L. Chang L. Hou Y. Guan S. (2018). Fabrication and characterization of biodegradable Mg-Zn-Y-Nd-Ag alloy: microstructure, mechanical properties, corrosion behavior and antibacterial activities. Bioact. Mater 3 (3), 225–235. 10.1016/j.bioactmat.2018.02.002 29744461 PMC5935780

[B28] Frazar E. M. Shah R. A. Dziubla T. D. Hilt J. Z. (2020). Multifunctional temperature-responsive polymers as advanced biomaterials and beyond. J. Appl. Polym. Sci. 137, 48770. 10.1002/app.48770 34305165 PMC8300996

[B29] Gao W. Han X. Sun D. Li Y. Liu X. Yang S. (2023). Antibacterial properties of antimicrobial peptide HHC36 modified polyetheretherketone. Front. Microbiol. 14, 1103956. 10.3389/fmicb.2023.1103956 36998411 PMC10043374

[B30] Garzoni C. Kelley W. L. (2011). Return of the trojan horse: intracellular phenotype switching and immune evasion by *Staphylococcus aureus* . EMBO Mol. Med. 3, 115–117. 10.1002/emmm.201100123 21365763 PMC3395108

[B31] Geng Z. Dong R. Li X. Xu X. Chen L. Han X. (2024). Study on the antibacterial activity and bone inductivity of Nanosilver/PLGA-Coated TI-CU implants. Int. J. Nanomedicine 19, 6427–6447. 10.2147/ijn.s456906 38952675 PMC11215459

[B32] Ghavimi S. Lungren E. S. Faulkner T. J. Josselet M. A. Wu Y. Sun Y. (2019). Inductive co-crosslinking of cellulose nanocrystal/chitosan hydrogels for the treatment of vertebral compression fractures. Int. J. Biol. Macromol. 130, 88–98. 10.1016/j.ijbiomac.2019.02.086 30779988

[B33] Han X. Sharma N. Xu Z. Krajewski S. Li P. Spintzyk S. (2024). A balance of biocompatibility and antibacterial capability of 3D printed PEEK implants with natural totarol coating. Dent. Mater 40 (4), 674–688. 10.1016/j.dental.2024.02.011 38388252

[B34] Hatashita S. Kawakami R. Ejiri S. Sasaki N. Toshiki N. Ito M. (2021). Acute masquelet technique for reconstructing bone defects of an open lower limb fracture. Eur. J. Trauma Emerg. Surg. 47, 1153–1162. 10.1007/s00068-019-01291-2 31894350

[B35] Hayashi K. Shimabukuro M. Zhang C. Taleb Alashkar A. N. Kishida R. Tsuchiya A. (2024). Silver phosphate-modified carbonate apatite honeycomb scaffolds for anti-infective and pigmentation-free bone tissue engineering. Mater Today Bio 27, 101161. 10.1016/j.mtbio.2024.101161 PMC11326936 39155941

[B36] He M. Wang H. Han Q. Shi X. He S. Sun J. (2023). Glucose-primed PEEK orthopedic implants for antibacterial therapy and safeguarding diabetic osseointegration. Biomaterials 303, 122355. 10.1016/j.biomaterials.2023.122355 37948855

[B37] Hou Z. Wang K. Liu G. Yuan Z. Peng H. Yuan Y. (2025). Nitric oxide-mediated dual-functional smart titanium implant coating for antibacterial and osseointegration promotion in implant-associated infections. Adv. Healthc. Mater 14, e2500997. 10.1002/adhm.202500997 40195820

[B38] Hu G. Wang H. Yao X. Bi D. Zhu G. Tang S. (2014). Development of nanofluorapatite polymer-based composite for bioactive orthopedic implants and prostheses. Int. J. Nanomedicine 9, 3875–3884. 10.2147/IJN.S65682 25143735 PMC4138000

[B39] Hu Q. Du Y. Bai Y. Xing D. Wu C. Li K. (2024). Smart zwitterionic coatings with precise pH-responsive antibacterial functions for bone implants to combat bacterial infections. Biomater. Sci. 12 (17), 4471–4482. 10.1039/d4bm00932k 39058335

[B40] Huang Y. Wan X. Su Q. Zhao C. Cao J. Yue Y. (2024). Ultrasound-activated piezo-hot carriers trigger tandem catalysis coordinating cuproptosis-like bacterial death against implant infections. Nat. Commun. 15 (1), 1643. 10.1038/s41467-024-45619-y 38388555 PMC10884398

[B41] Indrakumar S. Gugulothu S. B. Joshi A. Dash T. K. Mishra V. Tandon B. (2025). Silk composite-based multifunctional pellets for controlled release. Macromol. Biosci. 25 (2), e2400410. 10.1002/mabi.202400410 39427344

[B42] Janion C. (2008). Inducible SOS response system of DNA repair and mutagenesis in *Escherichia coli* . Int. J. Biol. Sci. 4 (6), 338–344. 10.7150/ijbs.4.338 18825275 PMC2556049

[B43] Ji Y. Yang S. Sun J. Ning C. (2023). Realizing both antibacterial activity and cytocompatibility in silicocarnotite bioceramic via germanium incorporation. J. Funct. Biomater. 14 (3), 154. 10.3390/jfb14030154 36976078 PMC10054726

[B44] Jia F. Guan J. Wang J. Li M. Zhang Y. Xie L. (2025). Zinc and melatonin mediated antimicrobial, anti-inflammatory, and antioxidant coatings accelerate bone defect repair. Colloids Surf. B Biointerfaces 245, 114335. 10.1016/j.colsurfb.2024.114335 39461184

[B45] Jin G. Qin H. Cao H. Qian S. Zhao Y. Peng X. (2014). Synergistic effects of dual Zn/Ag ion implantation in osteogenic activity and antibacterial ability of titanium. Biomaterials 35 (27), 7699–7713. 10.1016/j.biomaterials.2014.05.074 24947228

[B46] Jin Y. Liu H. Chu L. Yang J. Li X. Zhou H. (2024). Initial therapeutic evidence of a borosilicate bioactive glass (BSG) and Fe(3)O(4) magnetic nanoparticle scaffold on implant-associated staphylococcal aureus bone infection. Bioact. Mater 40, 148–167. 10.1016/j.bioactmat.2024.05.040 38962659 PMC11220464

[B47] Joshi M. U. Kulkarni S. P. Choppadandi M. Keerthana M. Kapusetti G. (2023). Current state of art smart coatings for orthopedic implants: a comprehensive review. Smart Mater. Med. 4, 661–679. 10.1016/j.smaim.2023.06.005

[B48] Josse J. Velard F. Gangloff S. C. (2015). *Staphylococcus aureus* vs. osteoblast: relationship and consequences in osteomyelitis. Front. Cell Infect. Microbiol. 5, 85. 10.3389/fcimb.2015.00085 26636047 PMC4660271

[B49] Kang B. J. Ryu H. H. Park S. S. Kim Y. Woo H. M. Kim W. H. (2012). Effect of matrigel on the osteogenic potential of canine adipose tissue-derived mesenchymal stem cells. J. Vet. Med. Sci. 74 (7), 827–836. 10.1292/jvms.11-0484 22313966

[B50] Kavanagh N. Ryan E. J. Widaa A. Sexton G. Fennell J. O'Rourke S. (2018). Staphylococcal osteomyelitis: disease progression, treatment challenges, and future directions. Clin. Microbiol. Rev. 31, e00084-17–00017. 10.1128/cmr.00084-17 PMC5967688 29444953

[B51] Kim H. K. DeDent A. Cheng A. G. McAdow M. Bagnoli F. Missiakas D. M. (2010). IsdA and IsdB antibodies protect mice against *Staphylococcus aureus* abscess formation and lethal challenge. Vaccine 28, 6382–6392. 10.1016/j.vaccine.2010.02.097 20226248 PMC3095377

[B52] Kintarak S. Whawell S. A. Speight P. M. Packer S. Nair S. P. (2004). Internalization of *Staphylococcus aureus* by human keratinocytes. Infect. Immun. 72, 5668–5675. 10.1128/iai.72.10.5668-5675.2004 15385465 PMC517534

[B53] Kobatake T. Miyamoto H. Hashimoto A. Ueno M. Nakashima T. Murakami T. (2019). Antibacterial activity of Ag-Hydroxyapatite coating against hematogenous infection by methicillin-resistant *Staphylococcus aureus* in the rat femur. J. Orthop. Res. 37 (12), 2655–2660. 10.1002/jor.24431 31373384

[B54] Kong P. Ren Y. Yang J. Fu W. Liu Z. Li Z. (2022). Relapsed boyhood tibia polymicrobial osteomyelitis linked to dermatophytosis: a case report. BMC Surg. 22 (1), 156. 10.1186/s12893-022-01600-4 35509041 PMC9066813

[B55] Krauss J. L. Roper P. M. Ballard A. Shih C. C. Fitzpatrick J. A. J. Cassat J. E. (2019). *Staphylococcus aureus* infects osteoclasts and replicates intracellularly. mBio 10, e02447-19–02419. 10.1128/mbio.02447-19 31615966 PMC6794488

[B56] Lai P. L. Chen L. H. Chen W. J. Chu I. M. (2013). Chemical and physical properties of bone cement for vertebroplasty. Biomed. J. 36, 162–167. 10.4103/2319-4170.112750 23989310

[B57] Lee H. Shin D. Y. Na Y. Han G. Kim J. Kim N. (2023). Antibacterial PLA/Mg composite with enhanced mechanical and biological performance for biodegradable orthopedic implants. Biomater. Adv. 152, 213523. 10.1016/j.bioadv.2023.213523 37336010

[B58] Li D. Li Y. Shrestha A. Wang S. Wu Q. Li L. (2019). Effects of programmed local delivery from a Micro/nano-hierarchical surface on titanium implant on infection clearance and osteogenic induction in an infected bone defect. Adv. Healthc. Mater 8, e1900002. 10.1002/adhm.201900002 30985090

[B59] Li D. Wei Q. Wu C. Zhang X. Xue Q. Zheng T. (2020). Superhydrophilicity and strong salt-affinity: zwitterionic polymer grafted surfaces with significant potentials particularly in biological systems. Adv. Colloid Interface Sci. 278, 102141. 10.1016/j.cis.2020.102141 32213350

[B60] Li J. Leung S. Chung Y. L. Chow S. K. H. Alt V. Rupp M. (2023). Hydrogel delivery of DNase I and liposomal vancomycin to eradicate fracture-related methicillin-resistant staphylococcus aureus infection and support osteoporotic fracture healing. Acta Biomater. 164, 223–239. 10.1016/j.actbio.2023.03.044 37019168

[B61] Li M. Liu J. Li Y. Chen W. Yang Z. Zou Y. (2024). Enhanced osteogenesis and antibacterial activity of dual-functional PEEK implants *via* biomimetic polydopamine modification with chondroitin sulfate and levofloxacin. J. Biomater. Sci. Polym. Ed. 35 (18), 2790–2806. 10.1080/09205063.2024.2390745 39155420

[B62] Li S. Yue Y. Wang W. Han M. Wan X. Li Q. (2024). Ultrasound-activated probiotics vesicles coating for titanium implant infections through bacterial Cuproptosis-Like death and immunoregulation. Adv. Mater 36 (44), e2405953. 10.1002/adma.202405953 39101293

[B63] Li Y. Liu C. Cheng X. Wang J. Pan Y. Liu C. (2023). PDA-BPs integrated mussel-inspired multifunctional hydrogel coating on PPENK implants for anti-tumor therapy, antibacterial infection and bone regeneration. Bioact. Mater 27, 546–559. 10.1016/j.bioactmat.2023.04.020 37397628 PMC10313727

[B64] Libraty D. H. Patkar C. Torres B. (2012). *Staphylococcus aureus* reactivation osteomyelitis after 75 years. N. Engl. J. Med. 366, 481–482. 10.1056/nejmc1111493 22296093 PMC3872831

[B65] Liu F. Wang X. Chen T. Zhang N. Wei Q. Tian J. (2020). Hydroxyapatite/silver electrospun fibers for anti-infection and osteoinduction. J. Adv. Res. 21, 91–102. 10.1016/j.jare.2019.10.002 32071777 PMC7015467

[B66] Liu Z. Yang L. Ni Y. Chen K. Yan Q. Zhao Z. (2024). Enhanced bacteriostasis and osseointegrative properties of SiRNA-modified polyetheretherketone surface for implant applications. PLoS One 19 (12), e0314091. 10.1371/journal.pone.0314091 39636795 PMC11620434

[B67] Majumdar U. Bose S. (2024). Curcumin and vitamin C dual release from hydroxyapatite coated Ti6Al4V discs enhances *in vitro* biological properties. Mater Chem. Phys. 313, 128622. 10.1016/j.matchemphys.2023.128622 38863477 PMC11164290

[B68] Masters E. A. Trombetta R. P. de Mesy Bentley K. L. Boyce B. F. Gill A. L. Gill S. R. (2019). Evolving concepts in bone infection: redefining “biofilm”, “acute vs. chronic osteomyelitis”, “the immune proteome” and local antibiotic therapy. Bone Res. 7, 20. 10.1038/s41413-019-0061-z 31646012 PMC6804538

[B69] Metsemakers W. J. Onsea J. Neutjens E. Steffens E. Schuermans A. McNally M. (2017). Prevention of fracture-related infection: a multidisciplinary care package. Int. Orthop. 41, 2457–2469. 10.1007/s00264-017-3607-y 28831576

[B70] Mohammed A. Saeed A. Elshaer A. Melaibari A. A. Memić A. Hassanin H. (2023). Fabrication and characterization of oxygen-generating polylactic acid/calcium peroxide composite filaments for bone scaffolds. Pharm. (Basel). 16 (4), 627. 10.3390/ph16040627 PMC10143609 37111384

[B71] Nagase K. (2021). Thermoresponsive interfaces obtained using poly(N-isopropylacrylamide)-based copolymer for bioseparation and tissue engineering applications. Adv. Colloid Interface Sci. 295, 102487. 10.1016/j.cis.2021.102487 34314989

[B72] Negi A. Verma A. Garg M. Goswami K. Mishra V. Singh A. K. (2024). Osteogenic citric acid linked chitosan coating of 3D-printed PLA scaffolds for preventing implant-associated infections. Int. J. Biol. Macromol. 282 (Pt 3), 136968. 10.1016/j.ijbiomac.2024.136968 39490474

[B73] Nelogi S. Kumarpatil A. Chowdhary R. Roy R. (2024). Optimising titanium implant stability and infection resistance through iron nanoparticle coatings: a preclinical investigation. J. Stomatol. Oral Maxillofac. Surg. 126 (6), 102155. 10.1016/j.jormas.2024.102155 39551183

[B74] Nguyen N. H. Lu Z. Elbourne A. Vasilev K. Roohani I. Zreiqat H. (2024). Engineering antibacterial bioceramics: design principles and mechanisms of action. Mater Today Bio 26, 101069. 10.1016/j.mtbio.2024.101069 PMC11099329 38765246

[B75] Ortiz-Benítez E. A. Velázquez-Guadarrama N. Durán Figueroa N. V. Quezada H. Olivares-Trejo J. J. (2019). Antibacterial mechanism of gold nanoparticles on Streptococcus pneumoniae. Metallomics 11, 1265–1276. 10.1039/c9mt00084d 31173034

[B76] Palla-Rubio B. Araújo-Gomes N. Fernández-Gutiérrez M. Rojo L. Suay J. Gurruchaga M. (2019). Synthesis and characterization of silica-chitosan hybrid materials as antibacterial coatings for titanium implants. Carbohydr. Polym. 203, 331–341. 10.1016/j.carbpol.2018.09.064 30318220

[B77] Patti J. M. Allen B. L. McGavin M. J. Höök M. (1994). MSCRAMM-mediated adherence of microorganisms to host tissues. Annu. Rev. Microbiol. 48, 585–617. 10.1146/annurev.micro.48.1.585 7826020

[B78] Pazarçeviren A. E. Dikmen T. Altunbaş K. Yaprakçı V. Erdemli Ö. Keskin D. (2020). Composite clinoptilolite/PCL-PEG-PCL scaffolds for bone regeneration: *in vitro* and *in vivo* evaluation. J. Tissue Eng. Regen. Med. 14, 3–15. 10.1002/term.2938 31475790

[B79] Piñera-Avellaneda D. Buxadera-Palomero J. Delint R. C. Dalby M. J. Burgess K. V. Ginebra M. P. (2024). Gallium and silver-doped titanium surfaces provide enhanced osteogenesis, reduce bone resorption and prevent bacterial infection in co-culture. Acta Biomater. 180, 154–170. 10.1016/j.actbio.2024.04.019 38621600

[B80] Porrino J. Wang A. Moats A. Mulcahy H. Kani K. (2020). Prosthetic joint infections: diagnosis, management, and complications of the two-stage replacement arthroplasty. Skelet. Radiol. 49, 847–859. 10.1007/s00256-020-03389-w 32040604

[B81] Qing Y. Li K. Li D. Qin Y. (2020). Antibacterial effects of silver incorporated zeolite coatings on 3D printed porous stainless steels. Mater Sci. Eng. C Mater Biol. Appl. 108, 110430. 10.1016/j.msec.2019.110430 31923959

[B82] Rennert-May E. D. Conly J. Smith S. Puloski S. Henderson E. Au F. (2018). The cost of managing complex surgical site infections following primary hip and knee arthroplasty: a population-based cohort study in Alberta, Canada. Infect. Control Hosp. Epidemiol. 39, 1183–1188. 10.1017/ice.2018.199 30196799

[B83] Sathiyavimal S. Vasantharaj S. Mattheos N. Pugazhendhi A. Subbalekha K. (2024). Mussel shell-derived biogenic hydroxyapatite as reinforcement on chitosan-loaded gentamicin composite for antibacterial activity and bone regeneration. Int. J. Biol. Macromol. 278 (Pt 2), 134143. 10.1016/j.ijbiomac.2024.134143 39069060

[B84] Satola S. W. Farley M. M. Anderson K. F. Patel J. B. (2011). Comparison of detection methods for heteroresistant vancomycin-intermediate *Staphylococcus aureus*, with the population analysis profile method as the reference method. J. Clin. Microbiol. 49, 177–183. 10.1128/jcm.01128-10 21048008 PMC3020420

[B85] Scialla S. Martuscelli G. Nappi F. Singh S. S. A. Iervolino A. Larobina D. (2021). Trends in managing cardiac and orthopaedic device-associated infections by using therapeutic biomaterials. Polym. (Basel) 13, 1556. 10.3390/polym13101556 PMC8151391 34066192

[B86] Shafiei M. R. Nezafati N. Karbasi S. Kharazi A. Z. (2025). Rifampin-loaded mesoporous silica nanoparticles improved physical and mechanical properties and biological response of acrylic bone cement. J. Med. Signals Sens. 15, 9. 10.4103/jmss.jmss_52_24 40191687 PMC11970834

[B87] Sheehy E. J. von Diemling C. Ryan E. Widaa A. O’ Donnell P. Ryan A. (2025). Antibiotic-eluting scaffolds with responsive dual-release kinetics facilitate bone healing and eliminate *S. aureus* infection. Biomaterials 313, 122774. 10.1016/j.biomaterials.2024.122774 39208699

[B88] Shen X. Zhang Y. Ma P. Sutrisno L. Luo Z. Hu Y. (2019). Fabrication of magnesium/zinc-metal organic framework on titanium implants to inhibit bacterial infection and promote bone regeneration. Biomaterials 212, 1–16. 10.1016/j.biomaterials.2019.05.008 31100479

[B89] Shi R. Ye J. Li W. Zhang J. Wu C. Xue J. (2019). Infection-responsive electrospun nanofiber mat for antibacterial guided tissue regeneration membrane. Mater Sci. Eng. C Mater Biol. Appl. 100, 523–534. 10.1016/j.msec.2019.03.039 30948089

[B90] Shi Y. Lai Y. Guo Y. Cai Z. Mao C. Lu M. (2024). Aspirin/amoxicillin loaded chitosan microparticles and polydopamine modified titanium implants to combat infections and promote osteogenesis. Sci. Rep. 14 (1), 7624. 10.1038/s41598-024-57156-1 38561345 PMC10984998

[B91] Shokri M. Kharaziha M. Tafti H. A. Eslaminejad M. B. Aghdam R. M. (2022). Synergic role of zinc and gallium doping in hydroxyapatite nanoparticles to improve osteogenesis and antibacterial activity. Biomater. Adv. 134, 112684. 10.1016/j.msec.2022.112684 35581072

[B92] Shu X. Liao J. Wang Q. Wang L. Shi Q. Xie X. (2024). Enhanced osteogenic and bactericidal performance of premixed calcium phosphate cement with photocrosslinked alginate thin film. J. Biomed. Mater Res. A 112 (7), 1057–1069. 10.1002/jbm.a.37688 38380877

[B93] Sun J. Li J. Shan A. Wang L. Ye J. Li S. (2025). A novel multifunctional PEEK internal fixation plate regulated by gentamicin/chitosan coating. Colloids Surf. B Biointerfaces 245, 114316. 10.1016/j.colsurfb.2024.114316 39405951

[B94] Sun L. Chen X. Ma K. Chen R. Mao Y. Chao R. (2023). Novel Titanium implant: a 3D multifunction Architecture with charge-trapping and piezoelectric self-stimulation. Adv. Healthc. Mater 12 (11), e2202620. 10.1002/adhm.202202620 36622654

[B95] Suo H. Li L. Zhang C. Yin J. Xu K. Liu J. (2020). Glucosamine-grafted methacrylated gelatin hydrogels as potential biomaterials for cartilage repair. J. Biomed. Mater Res. B Appl. Biomater. 108 (3), 990–999. 10.1002/jbm.b.34451 31369700

[B96] Szelerski Ł. Pajchert Kozłowska A. Żarek S. Górski R. Mochocki K. Dejnek M. (2021). A new criterion for assessing ilizarov treatment outcomes in nonunion of the tibia. Arch. Orthop. Trauma Surg. 141, 879–889. 10.1007/s00402-020-03571-8 32778920 PMC8049889

[B97] Tan J. Zhang H. Liu Y. Hou Z. Wang D. Zhou J. (2025). Interfering with proton and electron transfer enables antibacterial starvation therapy. Sci. Adv. 11 (12), eadt3159. 10.1126/sciadv.adt3159 40106542 PMC11922021

[B98] Tanadchangsaeng N. Pasanaphong K. Tawonsawatruk T. Rattanapinyopituk K. Tangketsarawan B. Rawiwet V. (2024). 3D bioprinting of fish skin-based gelatin methacryloyl (GelMA) bio-ink for use as a potential skin substitute. Sci. Rep. 14 (1), 23240. 10.1038/s41598-024-73774-1 39369014 PMC11455937

[B99] Tang X. Dai J. Sun H. Nabanita S. Petr S. Wang D. (2019). Mechanical strength, surface properties, cytocompatibility and antibacterial activity of nano zinc-magnesium silicate/polyetheretherketone biocomposites. J. Nanosci. Nanotechnol. 19 (12), 7615–7623. 10.1166/jnn.2019.16727 31196268

[B100] Teske S. S. Detweiler C. S. (2015). The biomechanisms of metal and metal-oxide nanoparticles' interactions with cells. Int. J. Environ. Res. Public Health 12, 1112–1134. 10.3390/ijerph120201112 25648173 PMC4344658

[B101] Uicich F. C. Merlo J. L. Redersdorff I. E. Herrera Seitz M. K. Pastore J. I. Ballarre J. (2024). Optimized electrophoretic deposition of chitosan/mesoporous glass nanoparticles with gentamicin on titanium implants: enhancing hemocompatibility and antibacterial activity. ACS Appl. Bio Mater 7 (7), 4642–4653. 10.1021/acsabm.4c00488 38967050

[B102] Valverde A. Pérez-Álvarez L. Ruiz-Rubio L. Pacha Olivenza M. A. García Blanco M. B. Díaz-Fuentes M. (2019). Antibacterial hyaluronic acid/chitosan multilayers onto smooth and micropatterned titanium surfaces. Carbohydr. Polym. 207, 824–833. 10.1016/j.carbpol.2018.12.039 30600071

[B103] Vu A. A. Robertson S. F. Ke D. Bandyopadhyay A. Bose S. (2019). Mechanical and biological properties of ZnO, SiO(2), and Ag(2)O doped plasma sprayed hydroxyapatite coating for orthopaedic and dental applications. Acta Biomater. 92, 325–335. 10.1016/j.actbio.2019.05.020 31082568

[B104] Wang J. Wang X. Liang Z. Lan W. Wei Y. Hu Y. (2023). Injectable antibacterial Ag-HA/GelMA hydrogel for bone tissue engineering. Front. Bioeng. Biotechnol. 11, 1219460. 10.3389/fbioe.2023.1219460 37388768 PMC10300446

[B105] Wang K. Rong F. Peng H. Yuan Z. Huo J. Liu P. (2024). Infection microenvironment-responsive coating on titanium surfaces for On-Demand release of therapeutic gas and antibiotic. Adv. Healthc. Mater 13 (18), e2304510. 10.1002/adhm.202304510 38532711

[B106] Wang X. Dong H. Liu J. Qin G. Chen D. Zhang E. (2019). *In vivo* antibacterial property of Ti-Cu sintered alloy implant. Mater Sci. Eng. C Mater Biol. Appl. 100, 38–47. 10.1016/j.msec.2019.02.084 30948074

[B107] Wang X. Pan L. Zheng A. Cao L. Wen J. Su T. (2023). Multifunctionalized carbon-fiber-reinforced polyetheretherketone implant for rapid osseointegration under infected environment. Bioact. Mater 24, 236–250. 10.1016/j.bioactmat.2022.12.016 36606257 PMC9803906

[B108] Wei W. Zhu J. Liu Y. Chen L. Ji H. Cheng Z. (2024). Graphene oxide-silver-coated sulfonated polyetheretherketone (Ag/GO-SPEEK): a broad-spectrum antibacterial artificial bone implants. ACS Appl. Bio Mater 7 (6), 3981–3990. 10.1021/acsabm.4c00338 38781457

[B109] Xie Q. Wang T. He L. Liang H. Sun J. Huang X. (2024). Biological and structural properties of curcumin-loaded graphene oxide incorporated collagen as composite scaffold for bone regeneration. Front. Bioeng. Biotechnol. 12, 1505102. 10.3389/fbioe.2024.1505102 39634102 PMC11614606

[B110] Yang D. Wijenayaka A. R. Solomon L. B. Pederson S. M. Findlay D. M. Kidd S. P. (2018). Novel insights into *Staphylococcus aureus* deep bone infections: the involvement of osteocytes. mBio 9, e00415–e00418. 10.1128/mbio.00415-18 29691335 PMC5915738

[B111] Yang J. Qin H. Chai Y. zhang P. Chen Y. Yang K. (2021). Molecular mechanisms of osteogenesis and antibacterial activity of Cu-bearing Ti alloy in a bone defect model with infection *in vivo* . J. Orthop. Transl. 27, 77–89. 10.1016/j.jot.2020.10.004 PMC7779545 33437640

[B112] Yao T. Chen J. Wang Z. Zhai J. Li Y. Xing J. (2019a). The antibacterial effect of potassium-sodium niobate ceramics based on controlling piezoelectric properties. Colloids Surf. B Biointerfaces 175, 463–468. 10.1016/j.colsurfb.2018.12.022 30572154

[B113] Yao T. T. Wang J. Xue Y. F. Yu W. j. Gao Q. Ferreira L. (2019b). A photodynamic antibacterial spray-coating based on the host-guest immobilization of the photosensitizer methylene blue. J. Mater Chem. B 7 (33), 5089–5095. 10.1039/c9tb01069f 31432872

[B114] Yu B. Pacureanu A. Olivier C. Cloetens P. Peyrin F. (2020). Assessment of the human bone lacuno-canalicular network at the nanoscale and impact of spatial resolution. Sci. Rep. 10, 4567. 10.1038/s41598-020-61269-8 32165649 PMC7067834

[B115] Yu Y. L. Wu J. J. Lin C. C. Qin X. Tay F. R. Miao L. (2023). Elimination of methicillin-resistant *Staphylococcus aureus* biofilms on titanium implants *via* photothermally-triggered nitric oxide and immunotherapy for enhanced osseointegration. Mil. Med. Res. 10 (1), 21. 10.1186/s40779-023-00454-y 37143145 PMC10158155

[B116] Yuan B. Chen H. Zhao R. Deng X. Chen G. Yang X. (2022). Construction of a magnesium hydroxide/graphene oxide/hydroxyapatite composite coating on mg-ca-zn-ag alloy to inhibit bacterial infection and promote bone regeneration. Bioact. Mater 18, 354–367. 10.1016/j.bioactmat.2022.02.030 35415306 PMC8965913

[B117] Zhang D. Liu W. Wu X. D. He X. Lin X. Wang H. (2019). Efficacy of novel nano-hydroxyapatite/polyurethane composite scaffolds with silver phosphate particles in chronic osteomyelitis. J. Mater Sci. Mater Med. 30, 59. 10.1007/s10856-019-6261-7 31127361

[B118] Zhang H. Zhang P. Shen X. Han J. Wang H. Qin H. (2025). A cross-linked coating loaded with antimicrobial peptides for corrosion control, early antibacterial, and sequential osteogenic promotion on a magnesium alloy as orthopedic implants. Acta Biomater. 193, 604–622. 10.1016/j.actbio.2024.12.046 39716540

[B119] Zhang L. Dai W. Gao C. Wei W. Huang R. Zhang X. (2023). Multileveled hierarchical hydrogel with continuous biophysical and biochemical gradients for enhanced repair of full-thickness osteochondral defect. Adv. Mater 35 (19), e2209565. 10.1002/adma.202209565 36870325

[B120] Zhang T. Zhou W. Yang W. Bi J. Li H. Gao X. (2024). Vancomycin-encapsulated hydrogel loaded microarc-oxidized 3D-printed porous Ti6Al4V implant for infected bone defects: reconstruction, anti-infection, and osseointegration. Bioact. Mater 42, 18–31. 10.1016/j.bioactmat.2024.07.035 39262845 PMC11388676

[B121] Zhang X. M. Li Y. Gu Y. X. Zhang C. N. Lai H. C. Shi J. Y. (2019). Ta-Coated titanium surface with superior bacteriostasis and osseointegration. Int. J. Nanomedicine 14, 8693–8706. 10.2147/ijn.s218640 31806965 PMC6842742

[B122] Zhao T. Liu X. Chu Z. Zhao J. Jiang D. Dong X. (2024). L-arginine loading porous PEEK promotes percutaneous tissue repair through macrophage orchestration. Bioact. Mater 40, 19–33. 10.1016/j.bioactmat.2024.05.025 38882001 PMC11179658

[B123] Zheng X. Luo H. Li J. Yang Z. Zhuan X. Li X. (2024). Zinc-doped bioactive glass-functionalized polyetheretherketone to enhance the biological response in bone regeneration. J. Biomed. Mater Res. A 112 (9), 1565–1577. 10.1002/jbm.a.37710 38514993

[B124] Zhou Q. Su X. Wu J. Zhang X. Su R. Ma L. (2023). Additive manufacturing of bioceramic implants for restoration bone engineering: technologies, advances, and future perspectives. ACS Biomater. Sci. Eng. 9 (3), 1164–1189. 10.1021/acsbiomaterials.2c01164 36786214

[B125] Zhuang Y. Zhang S. Yang K. Ren L. Dai K. (2020). Antibacterial activity of copper-bearing 316L stainless steel for the prevention of implant-related infection. J. Biomed. Mater Res. B Appl. Biomater. 108 (2), 484–495. 10.1002/jbm.b.34405 31074107

